# Microneedle-mediated co-delivery of biomimetic melanin and pro-regenerative factors for oxidative and immune modulation in vitiligo

**DOI:** 10.1016/j.mtbio.2026.103757

**Published:** 2026-10-17

**Authors:** Jingyao Liang, Wenyan Liu, Yu Jiang, Yihui Yu, Qian Li, Yuyan Zhang, Wei Li, Xibao Zhang

**Affiliations:** a Institute of Dermatology, Guangzhou Medical University, Guangzhou, 510095, PR China; b Department of Dermatology, Guangzhou Dermatology Hospital, Guangzhou, 510095, PR China

**Keywords:** Bionic melanin nanoparticles, Hydrogel microneedles, Vitiligo, Oxidative stress, Repigmentation

## Abstract

Vitiligo lesions are characterized by elevated epidermal oxidative stress, cytotoxic immune activation, and impaired melanogenesis, while conventional topical therapies often fail to modulate these pathogenic events due to limited skin penetration and therapeutic depth. To address these challenges, a dual-layer gelatin methacryloyl (GelMA)/hyaluronic acid methacryloyl (HAMA)//hyaluronic acid (HA) (Gel//HA) microneedle system was engineered. The inner HA layer rapidly dissolves upon insertion, releasing keratinocyte-membrane-coated artificial melanin nanoparticles (MPDA) to achieve early reactive oxygen species (ROS) scavenging and targeted delivery to keratinocytes. The outer Gel hydrogel layer enables sustained intradermal delivery of basic fibroblast growth factor (bFGF) and tyrosinase (TYR), supporting melanocyte survival and restoring melanin synthesis. The microneedles exhibited robust mechanical strength (0.234 ± 0.014 N/needle) with a penetration depth of approximately 300-350 μm. *In vitro*, the cumulative release of bFGF and TYR reached 65% and 70% within the first 72 h, respectively, and subsequently increased more gradually to 92.1% and 90.7% by day 21. *In vitro*, the combined administration of MPDA, bFGF, and TYR, mitigated H_2_O_2_-induced oxidative injury and enhanced keratinocyte viability. In a monobenzone-induced mouse model, Gel@bFGF/TYR//HA@MPDA treatment markedly promoted repigmentation, restored epidermal melanin deposition, and reduced the expression of CD8, IFN-γ, and TNF-α. Transcriptomic analysis further revealed suppression of ferroptosis-related genes and reactivation of the Wnt–Frizzled–MITF melanogenic signaling axis. This spatially designed microneedle system provides coordinated antioxidant, immunomodulatory, and pro-melanogenic effects, offering a promising localized therapeutic strategy for vitiligo.

## Graphical abstract

**Figure:**
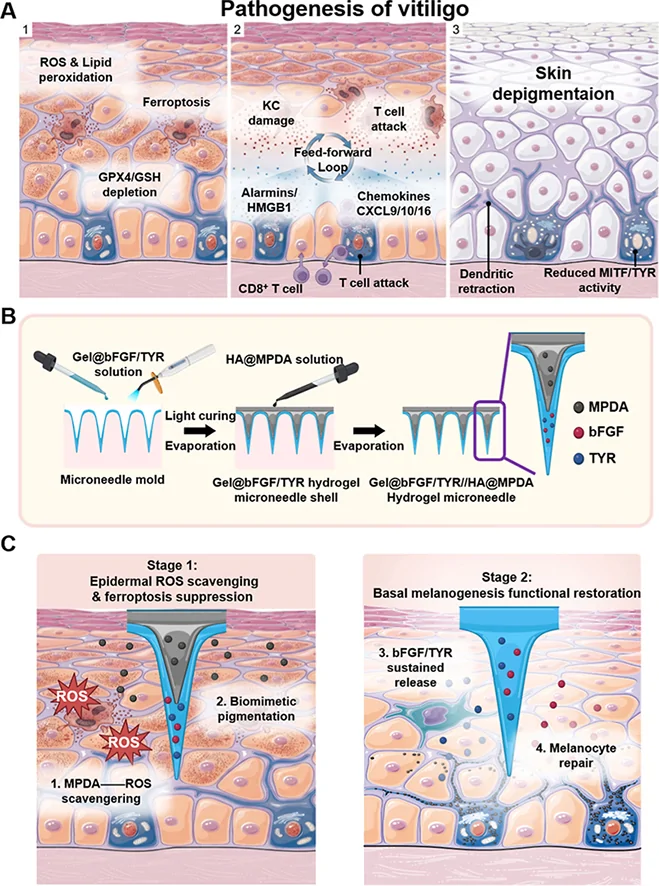

## 1 Introduction

Vitiligo is a multifactorial depigmenting disorder of the epidermis, affecting approximately 1-2% of the global population and significantly impairing appearance and quality of life [1], [2]. The pathogenesis of vitiligo is not solely attributable to melanocyte loss; rather, it arises from the combined effects of oxidative imbalance, immune-mediated epithelial injury, and long-term suppression of the melanogenic network [1], [3], [4]. Substantial evidence indicates that keratinocytes (KCs) experience pronounced oxidative stress in the early stages of vitiligo. During this period, intrinsic antioxidant defenses, such as glutathione peroxidase 4 (GPX4), glutathione (GSH), and ferritin heavy chain 1 (FTH1), are depleted, accompanied by acyl-CoA synthetase long-chain family member 4 (ACSL4)-mediated lipid peroxidation and ferroptosis activation, resulting in heightened KC vulnerability to external stimuli [5], [6], [7], [8], [9], [10], [11]. Damaged KCs subsequently release alarmins such as high mobility group box 1 (HMGB1) and induce high expression of chemokines CXCL9/10/16, thereby forming a chemotactic gradient that recruits CD8^+^ T cells into the epidermis and amplifies local cytotoxicity [12], [13], [14]. This “oxidative stress–chemokine–CD8^+^ T cell” feed-forward loop is regarded as a central driver of lesion expansion.

Under persistent oxidative and immune stress, basal melanocytes develop multiple functional impairments, including reduced microphthalmia-associated transcription factor (MITF) activity, insufficient expression of tyrosinase family enzymes, disrupted melanosome maturation, and impaired KC–melanocyte communication. Although melanosomes are synthesized within melanocytes, their transfer to KCs and subsequent deposition in the suprabasal epidermis are equally essential for visible repigmentation [15], [16]. In vitiligo lesions, elevated oxidative stress and damaged KC–melanocyte (MC) interfaces hinder both melanin synthesis and melanosome transport, leading to unstable and incomplete repigmentation even after inflammation is controlled [3], [4], [17], [18]. Current treatments—including JAK inhibitors, corticosteroids, and phototherapy—primarily target immunologic or inflammatory pathways, but fail to simultaneously restore early epidermal oxidative imbalance and the later melanosome synthesis–transfer chain, resulting in suboptimal repigmentation and high recurrence rates [19], [20], [21]. Therefore, directly supplying biomimetic pigment particles to KCs while reactivating melanogenic signaling in deeper layers has emerged as a promising direction in next-generation repigmentation materials.

Among artificial pigment materials, polydopamine (PDA) has gained particular attention due to its structural similarity to natural eumelanin, exhibiting strong free-radical scavenging capacity, broad-spectrum light absorption, and favorable biocompatibility [22], [23]. Coating PDA nanoparticles with KC membranes further enhances their affinity and uptake by KCs, creating a delivery mode analogous to physiological melanosome transfer. Compared with other cell-derived membranes (e.g., erythrocytes, macrophages), KC membranes preserve native epidermal adhesion molecules and interface characteristics, such as E-cadherin and integrin families, which facilitate retention and internalization within the epidermis [24], [25]. Moreover, PDA shares a melanotic optical absorption profile and can form stable intracellular deposits, contributing directly to visible pigmentation at the tissue level [10], [26]. Thus, KC-membrane-coated PDA nanoparticles (MPDA) can function both as local antioxidants and as biomimetic pigment donors aligned with the epidermal requirements of vitiligo repigmentation.

Despite these advantages, topical or injectable administration of MPDA alone faces significant limitations [27], [28]. The stratum corneum forms an almost impermeable barrier to nanoparticles, preventing efficient access to KC layers even with homotypic membrane coating. Conversely, subcutaneous injection deposits particles too deeply within the dermis, creating spatial mismatch with their intended epidermal target. In addition, the disrupted epidermal barrier and unstable microenvironment in vitiligo lesions promote nonspecific diffusion or phagocytic clearance of free nanoparticles, limiting local retention and color deposition. Importantly, MPDA must be accurately delivered to and retained within KC layers to produce visible pigmentation; otherwise, their biomimetic potential cannot be fully realized.

To achieve effective local delivery of MPDA and protein therapeutics, the limitations of conventional hydrogel-based approaches must be considered. Topically applied hydrogels can improve skin hydration and local residence time but generally remain on the skin surface and do not independently overcome the barrier imposed by the stratum corneum. Injectable hydrogels can bypass this barrier and provide local drug retention; however, they typically form a bulk depot at the injection site and offer limited control over shallow delivery across the epidermal and dermal regions. In contrast, hydrogel microneedles integrate the drug-retaining properties of hydrogels with the physical penetration capability of microscale needles. By creating controlled microchannels through the stratum corneum, they facilitate the delivery of nanoparticles and macromolecular agents into viable skin while reducing the tissue disruption associated with conventional injection [29], [30], [31], [32]. Moreover, the geometry and material composition of microneedles can be independently adjusted to regulate penetration depth, hydration behavior, and release kinetics. These features make microneedles particularly suitable for vitiligo treatment, in which different therapeutic components must be delivered across the skin barrier and released at different rates to address the spatially distinct pathological processes within the lesion.

Building on these advantages and considering the spatially distinct pathological changes within vitiligo lesions, we designed a dual-layer GelMA/HAMA//HA microneedle system in which the two compartments perform complementary mechanical and drug-release functions. The inner microneedle layer consists of rapidly dissolving HA, loaded with MPDA. Following skin penetration and microchannel formation, interstitial fluid reaches the non-crosslinked HA compartment, leading to its rapid hydration and dissolution. The encapsulated MPDA is consequently released into the fluid-filled microchannels and becomes available to the epidermal microenvironment to address early ROS accumulation and ferroptosis-related oxidative stress. MPDA, comprising a PDA core coated with KC membrane, provides enhanced KC uptake and potent free-radical scavenging activity [10], thereby exerting local antioxidative modulation and supplying biomimetic pigment directly to the epidermis. The outer layer is composed of a highly cross-linked GelMA/HAMA hydrogel network, designed to load and sustain the release of basic fibroblast growth factor (bFGF) and tyrosinase (TYR). These factors support melanocyte survival, supplement TYR activity, and maintain MITF signaling—processes that require extended local availability to gradually restore melanogenic function. The three-dimensional GelMA/HAMA matrix maintains prolonged drug retention, enabling simultaneous early KC-level oxidation control and deeper melanocyte functional recovery within a single microneedle insertion (Scheme 1).

**Scheme 1:**
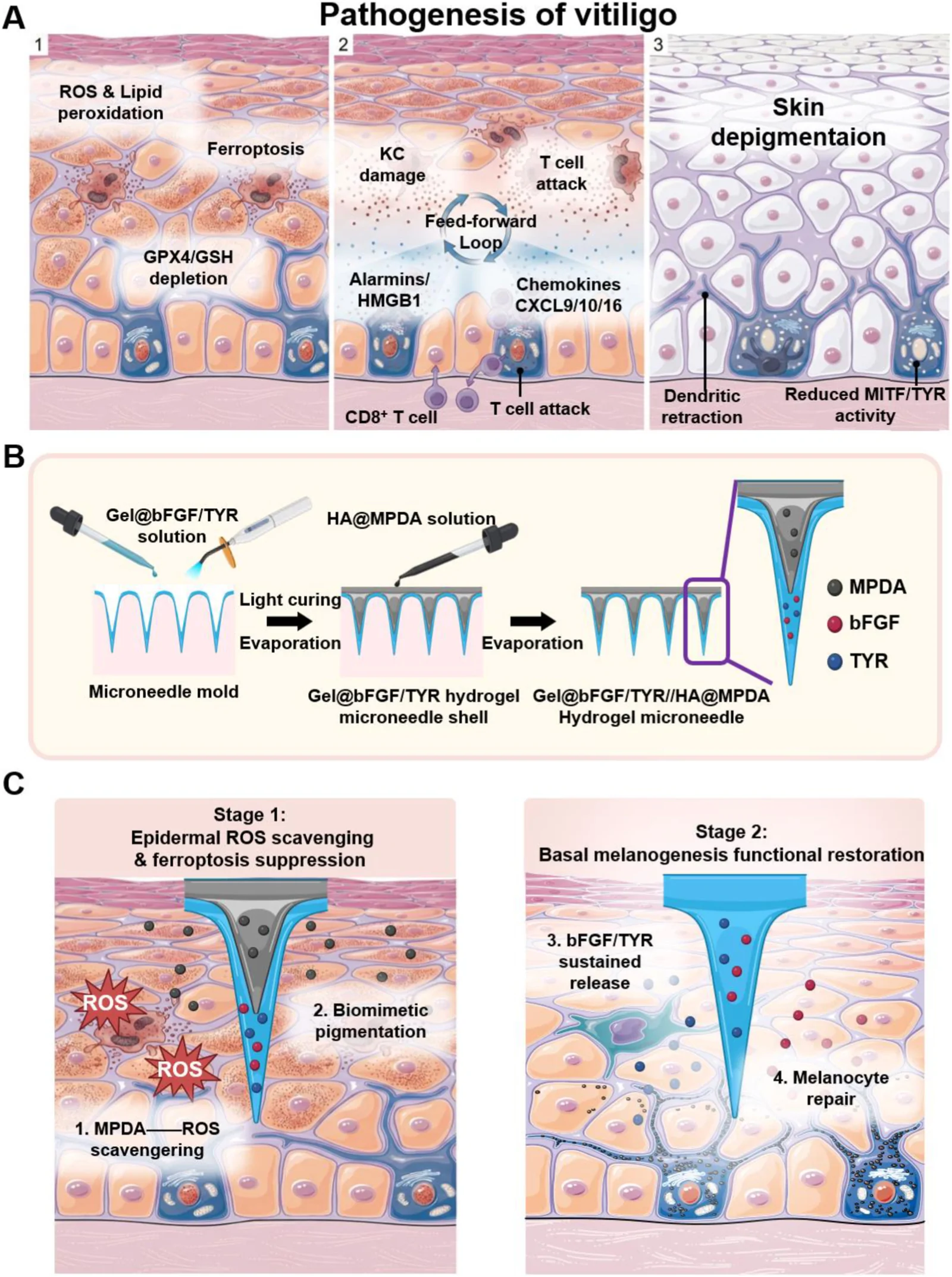
**Schematic diagram of the pathogenesis of vitiligo and microneedle treatment.** (A) Pathogenesis of vitiligo; (B) Microneedle preparation process and bilayer structure; (C) Schematic illustration of the intradermal delivery and therapeutic mechanisms of the bilayer microneedles.

In this study, we systematically characterized the structural integrity, mechanical performance, drug loading, and release behavior of the dual-layer microneedles. Furthermore, we evaluated their biological effects using an *in vitro* ROS-induced injury model, an *in vivo* monobenzone-induced vitiligo-like mouse model, and transcriptomic analysis to assess regulation of oxidative stress, ferroptosis, immune activation, and melanogenesis pathways. Collectively, this work establishes a mechanistically oriented, lesion-layer-matched microneedle delivery strategy that offers a structurally feasible, biologically rational, and multi-target synergistic platform for topical vitiligo therapy.

## 2 Materials and methods

### 2.1 Materials

Gelatin from porcine skin was obtained from Sigma–Aldrich. Sodium hyaluronate and tyrosinase (TYR; biochemical reagent grade, 500 U/mg) were purchased from Shanghai Yuanye Bio-Technology Co., Ltd. (Shanghai, China). Poly(vinyl alcohol) 1788, hydrogen peroxide (H_2_O_2_), rhodamine B (Rho B), fluorescein isothiocyanate (FITC), and the ABTS and DPPH radical-scavenging assay kits were purchased from Macklin Biochemical Co., Ltd. (Shanghai, China). Methacrylic anhydride (94%), dopamine hydrochloride, and lithium phenyl-2,4,6-trimethylbenzoylphosphinate (LAP) were obtained from Aladdin Biochemical Technology Co., Ltd. (Shanghai, China). Recombinant basic fibroblast growth factor (bFGF) was purchased from Cloud-Clone Corp. (Wuhan, China), and the bFGF ELISA kit was obtained from Jiangsu Meimian Industrial Co., Ltd. (Jiangsu, China). The DCFH-DA assay kit, CCK-8 assay kit, and cell membrane protein extraction kit were purchased from Beyotime Biotechnology (Shanghai, China). The live/dead cell staining kit was obtained from KeyGEN Biotech Co., Ltd. (Jiangsu, China). DAPI and the total superoxide dismutase (T-SOD), catalase (CAT), glutathione (GSH), and malondialdehyde (MDA) assay kits were purchased from Wuhan Servicebio Technology Co., Ltd. (Wuhan, China). TRIzol reagent was also obtained from Wuhan Servicebio Technology Co., Ltd. Primary antibodies against Ki67, IFN-γ, TNF-α, CD8, SOD2, CAT, GPX4, 4-HNE, FTH1, PTGS2, TRP1, TRP2, MITF, and GAPDH, together with the corresponding secondary antibodies, were purchased from Proteintech Group, Inc. (Wuhan, China). Dulbecco's modified Eagle's medium (DMEM), penicillin–streptomycin, 0.25% trypsin, and fetal bovine serum were obtained from Gibco (Shanghai, China). Commercial 0.1% tacrolimus ointment (Protopic®) was used as the positive control in the animal experiments. All other reagents were of analytical grade and used as received without further purification.

### 2.2 Preparation of PDA and MPDA

The synthesis of PDA was the same as that of previous reported methods [33]. Dopamine hydrochloride was added to a tris buffer solution (0.05 M, pH 8.5) and the reaction was stirred for 6 h at room temperature. Salts and impurities were removed via dialysis using a cellulose dialysis bag with a molecular weight cut-off of 1200 Da in deionized water. PDA was finally obtained by freeze-drying and stored at −20°C until use. For preparing MPDA, HaCaT cell membrane solution was mixed with PDA and sonicated well, and the mixture was co-extruded through a polycarbonate porous membrane 11 times. The solution was washed 3 times with deionized water at 4°C (13000 rpm, 10 min) to obtain MPDA.

### 2.3 Synthesis of GelMA

GelMA was prepared using a previously reported method with minor modifications [31]. The Gelatin was dissolved in distilled water and methacrylic anhydride was added to react for 1 h at 50°C. Salts and impurities were dialyzed in deionized water using a cellulose dialysis bag with a molecular weight cut-off of 3500 Da. GelMA was finally obtained by freeze-drying.

### 2.4 Synthesis of HAMA

The synthesis of HAMA was the same as that of previous reported methods [34]. HA was dissolved in distilled water, then methacrylic anhydride was added and the pH was adjusted to between 8 and 9. The reaction was stirred at 4°C for 24 h. Salts and impurities were dialyzed in deionized water using a cellulose dialysis bag with a molecular weight cut-off of 12000 Da. HAMA was finally obtained by freeze-drying.

### 2.5 Preparation of GelMA/HAMA//HA and GelMA/HAMA@bFGF/TYR//HA/MPDA separable hydrogel microneedles

#### 2.5.1 Preparation of precursor solutions

Poly(vinyl alcohol) 1788 (PVA, 20%, w/v) and HA (3%, w/v) were separately dissolved in deionized water. GelMA (10%, w/v) and HAMA were dissolved in PBS containing LAP (0.2%, w/v) to prepare the photocrosslinkable outer-layer precursor. The HAMA concentration was adjusted to 2%, 5%, or 10% (w/v), yielding GelMA-2-HAMA, GelMA-5-HAMA, and GelMA-10-HAMA formulations, respectively. Unless otherwise specified, GelMA-10-HAMA was used for the subsequent preparation of the bilayer microneedles.

For the drug-loaded formulations, TYR and bFGF were directly incorporated into the GelMA/HAMA precursor solution at final concentrations of 1 mg/mL and 1000 ng/mL, respectively. Based on the specific activity of TYR (500 U/mg), the TYR concentration corresponded to 500 U/mL. MPDA was dispersed in the 3% (w/v) HA solution at a final concentration of 100 μg/mL. The resulting GelMA/HAMA solution containing bFGF and TYR and the HA solution containing MPDA were used to prepare the crosslinked outer layer and non-crosslinked dissolving compartment, respectively.

#### 2.5.2 Fabrication of bilayer hydrogel microneedles

The bilayer hydrogel microneedles were fabricated using a sequential casting, photocrosslinking, and drying method. Briefly, 100 μL of GelMA/HAMA precursor solution, with or without bFGF and TYR, was added to each silicone mold containing a 10 × 10 microneedle array. Negative pressure was applied to remove trapped air and facilitate contact between the precursor solution and the walls of the microneedle cavities. After excess solution was removed from the mold surface, the GelMA/HAMA layer was photocrosslinked by exposure to 405 nm light at 35 mW/cm^2^ for 30 s.

The photocrosslinked GelMA/HAMA layer was then completely dried at 4°C. During drying, removal of water caused substantial volume contraction of the hydrogel, leaving a thin crosslinked GelMA/HAMA layer along the microneedle cavities rather than filling the entire needle volume. This dried layer subsequently served as the mechanically supportive outer compartment of the microneedles.

Next, 500 μL of 3% (w/v) HA solution, with or without MPDA, was added to the mold. Negative pressure was applied again to allow the HA solution to enter and fill the remaining space within the microneedle cavities. The mold was then dried at 4°C until the HA compartment was completely solidified. Because HA was not photocrosslinked, this compartment remained rapidly dissolvable upon exposure to interstitial fluid.

Finally, 20% (w/v) PVA solution was added and dried at 4°C to form a supporting backing layer, facilitating handling and application of the microneedle patches. After complete drying, the patches were carefully removed from the molds. The resulting formulations were designated Gel//HA, Gel@bFGF/TYR//HA, Gel//HA@MPDA, and Gel@bFGF/TYR//HA@MPDA. All fabricated microneedles were stored in a desiccator at 4°C until further use.

### 2.6 Morphology and size distribution characterization of PDA and MPDA

PDA and MPDA were dispersed in deionized water after sonication and added dropwise onto a copper mesh, which was left to dry completely before being photographed morphologically. The morphology of PDA and MPDA was observed by transmission electron microscopy (TEM, CEM 902A, Zeiss, Germany). The PDA and MPDA suspensions were placed in potentiometric dishes and their hydrated particle size was examined using a nano-laser particle sizer (DLS, Nano ZS, Malvern, UK), cycling the test at least three times.

### 2.7 Characterization of GelMA and HAMA

#### 2.7.1 Chemical structure characterization of GelMA and HAMA

The chemical structure of GelMA, HAMA, gelatin, and HA were characterized by ^1^H NMR (Inova-500 M, Varian, USA) spectroscopy. For ^1^H NMR characterization, 8 mg of samples was totally dissolved in deuterated water (D_2_O).

#### 2.7.2 Rheological characterization of GelMA/HAMA hydrogels

The rheological properties of GelMA-2-HAMA, GelMA-5-HAMA, and GelMA-10-HAMA hydrogels were evaluated using a rotational rheometer (Kinexus Pro, Malvern, UK) at 37°C. Time-sweep measurements were performed at a fixed frequency of 1 Hz and a strain of 1%. Frequency-sweep measurements were subsequently conducted over a frequency range of 1–100 Hz at a constant strain of 1%. The storage modulus (G′) and loss modulus (G″) were recorded to evaluate the viscoelastic properties of the different hydrogel formulations.

#### 2.7.3 Compression test of the cylindrical GelMA and HAMA hydrogel

GelMA-2-HAMA, GelMA-5-HAMA, and GelMA-10-HAMA hydrogels were prepared to a cylindrical shape with a diameter of 11 mm and a height of 6 mm. A universal testing machine was used to record the displacement and pressure of the hydrogels. The probe was pressed down vertically at a constant speed of 0.05 mm/s, and the displacement and pressure were recorded in real time. The compression strength was calculated by the following formula:

Compressionstrength(Pa)=F/S

Where F is the pressure recorded by the sensor and S is the cross-sectional area of the cylinder. Strain is the ratio of compressive displacement to the original height of the cylinder. Curves were drawn from compressive strength and strain to reflect the change in the compressive strength of the hydrogel.

#### 2.7.4 *In vitro* swelling test

Gel-2-HAMA, Gel-5-HAMA, and Gel-10-HAMA hydrogels were firstly weighted, then immersed in PBS buffer. After immersed for 1, 2, 4, 6, and 24 h, the hydrogel was taken out and excess water on the hydrogel was carefully wiped off. The initial weight and swollen weight were recorded as M_0_ and M_t_, respectively.

$$\text{Swelling}\;\text{ratio}\;\left(\%\right)=M_{t}/M_{0}\times 100\%$$

### 2.8 Characterization of GelMA/HAMA//HA hydrogel microneedles

#### 2.8.1 Morphology characterization of HAMA/GelMA//HA hydrogel microneedles

The appearance of the hydrogel microneedles was photographed by an inverted fluorescence microscopy and scanning electron microscopy (SEM). For capturing microneedles with fluorescence, rhodamine B (Rho B) and fluorescein isothiocyanate (FITC) were used to label HA and GelMA, respectively.

#### 2.8.2 Mechanical strength of bilayer hydrogel microneedles

The mechanical strength of the bilayer hydrogel microneedles was measured using a universal testing machine. Each microneedle patch, consisting of a 10 × 10 needle array, was placed with the needle tips facing upward. A flat compression probe was moved vertically toward the array at a constant rate of 0.05 mm/s until a displacement of 0.4 mm was reached. The total compressive force and displacement were recorded continuously. The mechanical strength per needle was calculated by dividing the total force measured at 0.4 mm displacement by the 100 needles in each array.

#### 2.8.3 *In vitro* puncture experiment

Fresh porcine skin was obtained from the market, and the micro-needles were thoroughly dried and inserted vertically into the porcine skin. After removing the microneedle, the porcine skins punctured by microneedles were fixed with 4% paraformaldehyde for 24 h, then embedded in paraffin and sectioned for H&E staining. To take three-dimensional (3D) confocal layer scan images, Rho B (1 mg/mL) was first added to the GelMA/HAMA mixture solution. Then, the solutions were added according to the steps for preparing GelMA/HAMA//HA hydrogel microneedles. The completely dried GelMA/HAMA//HA microneedles were pierced into the porcine skin. Scanning images were acquired at intervals of 10 μm with a confocal laser microscope, and the images were reconstructed in 3D.

### 2.9 Loading and *in vitro* release characterization of MPDA, bFGF, and TYR

#### 2.9.1 Determination of bFGF and TYR loading amounts

The loading amounts of bFGF and TYR were determined by measuring the residual agents remaining on the mold surface after microneedle fabrication. For each 10 × 10 microneedle array, 100 μL of GelMA/HAMA precursor solution containing bFGF (1000 ng/mL) and TYR (1 mg/mL) was cast into the mold, corresponding to initial input amounts of 100 ng and 100 μg, respectively. After photocrosslinking and drying, the mold surface was rinsed with 100 μL of PBS to recover the residual non-encapsulated agents. The bFGF concentration in the washing solution was measured using an ELISA kit after appropriate dilution, whereas the TYR concentration was determined by UV–Vis spectrophotometry at 280 nm using a TYR calibration curve.

#### 2.9.2 MPDA release from bilayer microneedles

One Gel//HA@MPDA microneedle patch was immersed in 2 mL of PBS at 37°C under continuous shaking. During the first 5 min, 1 mL of release medium was collected at 1-min intervals and immediately replaced with an equal volume of fresh PBS. The MPDA concentration in each sample was determined by UV–Vis spectrophotometry at 390 nm using an MPDA calibration curve. The cumulative release percentage was calculated relative to the initial amount of MPDA incorporated into the microneedle patch.

#### 2.9.3 Release of bFGF and TYR from bulk GelMA/HAMA hydrogels

Because the amounts of bFGF and TYR in an individual microneedle patch were insufficient for reliable long-term quantification, bulk GelMA/HAMA hydrogels with the same composition and photocrosslinking conditions as the microneedle outer layer were used for release measurements. Briefly, bFGF and TYR were incorporated into 1 mL of GelMA/HAMA precursor solution at final concentrations of 1000 ng/mL and 1 mg/mL, respectively. The precursor was photocrosslinked under 405 nm light at 35 mW/cm^2^ for 30 s and then immersed in 2 mL of PBS at 37°C.

At each predetermined time point, the entire release medium was collected and replaced with 2 mL of fresh PBS. The released bFGF was quantified using an ELISA kit, while TYR was quantified by UV–Vis spectrophotometry at 280 nm.

To evaluate the enzymatic activity of TYR after release, the TYR concentration in the release medium collected at 72 h was first determined. Free TYR was then diluted to the same concentration and used as the reference. TYR activity was measured using a commercial tyrosinase activity assay kit according to the manufacturer's instructions. Release medium from blank GelMA/HAMA hydrogels was used as the background control, and the activity of released TYR was expressed relative to that of free TYR at the same concentration.

### 2.10 Cellular uptake of PDA and MPDA by HaCaT cells

HaCaT cells were seeded into 24-well plates at a density of 5 × 10^4^ cells per well and cultured for 24 h. PDA and MPDA were labeled with Rho B to obtain Rho-PDA and Rho-MPDA, respectively. The culture medium was then replaced with complete high-glucose DMEM containing Rho-PDA or Rho-MPDA. After incubation for 1, 2, or 4 h, the cells were washed with PBS to remove free nanoparticles.

For fluorescence imaging, the cells were fixed with 4% paraformaldehyde, and the nuclei were counterstained with DAPI. Cellular uptake of Rho-PDA and Rho-MPDA was observed using a fluorescence microscope. For quantitative analysis, the cells were collected after the indicated incubation periods, washed with PBS, and analyzed by flow cytometry. The mean intracellular Rho B fluorescence intensity was quantified using FlowJo software.

### 2.11 *In vitro* cytotoxicity assay

Cytotoxicity of GelMA/HAMA//HA hydrogels was examined using human immortalized epidermal HaCaT cells. HaCaT cells were cultured in a DMEM high glucose medium to a logarithmic growth phase, digested with trypsin, resuspended in a DMEM high glucose medium, and seeded in 96-well plates or 24-well plates. GelMA/HAMA//HA (Gel//HA), GelMA/HAMA@bFGF/TYR//HA (Gel@bFGF/TYR//HA), GelMA/HAMA//HA/MPDA (Gel//HA@MPDA) and GelMA/HAMA@

bFGF/TYR//HA@MPDA (Gel@bFGF/TYR//HA@MPDA) were weighed and then immersed in a DMEM high-glucose medium (the mass ratio of the hydrogel to the medium was 1:10) and leached at a constant temperature of 37°C for 24 h. The leached stock solution was directly used for subsequent cell culture. After the cells inoculated on the 96-well plate or 24-well plate adhered for 24 h, the original medium was replaced with the hydrogel extract, and the culture was continued. Cell viability was detected with a CCK-8 kit after 1 and 3 days of culture in 96-well plates, respectively. For Ki67 immunofluorescence staining, cells cultured in 24-well plates were used for detection and labeling of proliferating cells.

### 2.12 *In vitro* scratch assay

HaCaT cells were seeded into 24-well plates at a density of 20000 cells per well and cultured for 24 h until a confluent monolayer was formed. A linear scratch was created in the center of each well using a sterile 200 μL pipette tip. The wells were gently washed twice with PBS to remove detached cells and cellular debris. The culture medium was then replaced with extracts obtained from Gel//HA, Gel@bFGF/TYR//HA, Gel//HA@MPDA, or Gel@bFGF/TYR//HA@MPDA. Cells cultured in an equivalent volume of high-glucose DMEM served as the control group.

Images of the same predefined fields were captured at 0 h and at the indicated time points (6 h, 24 h and 48 h]) using an inverted microscope under identical imaging conditions. The cell-free scratch area was quantified using ImageJ software. Scratch closure was calculated according to the following equation:

$$\text{Scratch}\;\text{closure}\;\left(\%\right)=\left[\left(A_{0}-A_{t}\right)/A_{0}\right]\times 100\%$$

where A_0_ represents the initial scratch area at 0 h and A_t_ represents the remaining scratch area at the indicated time point.

### 2.13 *In vitro* ROS injured cell protection experiment

HaCaT cells were injured with a H_2_O_2_ solution to establish an ROS injury model. The cells were treated with 0, 200, 400, 800, 1200, 1600, 2000, and 2400 μmol/L H_2_O_2_ solution for 6 h, and then the cell viability was detected by the CCK-8 method. The H_2_O_2_ concentration at which the cell viability was 50% after injury was selected as the final concentration for establishing the ROS injury model. After the injured cells were further cultured with the Gel//HA, Gel@bFGF/TYR//HA, Gel//HA@MPDA, and the Gel@bFGF/TYR//HA@MPDA leached stock solution for 24 h, the cell viability was detected by the CCK-8 kit to evaluate the protective effect on the HaCaT cells after ROS injury. Live and dead staining kits were used to stain cells, labeling live (green) and dead (red) cells separately. At the same time, ROS fluorescence intensity was detected with the DCFH fluorescent probe.

### 2.14 Evaluation of oxidative injury and melanin-associated responses in B16-F10 cells

#### 2.14.1 Cell culture and cytocompatibility

B16-F10 cells were purchased from Guangzhou Yuanjing Biotechnology Co., Ltd (Guangzhou, China) and cultured in RPMI-1640 medium supplemented with 10% fetal bovine serum and 1% penicillin–streptomycin at 37°C under 5% CO_2_. Cells were detached using 0.05% trypsin for subsequent experiments.

For cytocompatibility evaluation, B16-F10 cells were seeded into 24-well plates at a density of 1 × 10^4^ cells per well and cultured overnight. The cells were subsequently treated with bFGF/TYR (200 ng/mL bFGF and 200 U/mL TYR), MPDA (100 μg/mL), or their combination. Untreated cells served as the control. Cell viability was determined after 1, 2, and 3 days using a CCK-8 assay according to the manufacturer's instructions.

#### 2.14.2 H_2_O_2_-induced oxidative injury

B16-F10 cells were exposed to 1600 μmol/L H_2_O_2_ for 6 h to induce oxidative injury. The H_2_O_2_-containing medium was then removed, and the cells were washed twice with PBS. The injured cells were subsequently cultured with extracts of Gel//HA, Gel@bFGF/TYR//HA, Gel//HA@MPDA, or Gel@bFGF/TYR//HA@MPDA. Untreated cells and H_2_O_2_-injured cells without extract treatment were used as the control and model groups, respectively. Unless otherwise specified, the cells were treated with the microneedle extracts for 24 h before subsequent analyses. Cell viability during recovery was evaluated using a CCK-8 assay on days 1, 2, and 3. The microneedle extracts were prepared as described in Section 2.11.

#### 2.14.3 Intracellular ROS and biochemical analyses

After treatment, intracellular ROS levels were measured using a DCFH-DA assay kit according to the manufacturer's instructions. For fluorescence imaging, the stained cells were observed using a fluorescence microscope, and the fluorescence intensity was quantified using ImageJ software. For flow cytometric analysis, the stained cells were collected, washed with PBS, and analyzed using a flow cytometer. The mean DCFH-DA fluorescence intensity was quantified using FlowJo software.

Treated cells were collected and lysed for biochemical analyses. T-SOD activity, CAT activity, GSH content, and MDA content were determined using the corresponding assay kits according to the manufacturers’ instructions. The results were normalized to the total protein content of each cell lysate. T-SOD and CAT activities were expressed as U/mg protein, whereas GSH and MDA levels were expressed as μmol/g protein.

#### 2.14.4 Determination of melanin-associated content

B16-F10 cells were seeded into 24-well plates at a density of 1 × 10^4^ cells per well and cultured overnight. For the cytocompatibility experiment, the cells were treated with bFGF/TYR, MPDA, or their combination for 24 h. For the oxidative injury experiment, H_2_O_2_-injured cells were treated with the different microneedle extracts for 24 h.

After treatment, the culture medium was removed, and the cells were washed three times with PBS to remove extracellular MPDA and residual medium. Each well was then treated with 50 μL of 1 mol/L NaOH for 5–10 min. The cell lysates were collected and heated at 80°C for 2 h.

Water-soluble melanin dissolved in 1 mol/L NaOH was used to prepare standards at concentrations of 0, 6.25, 12.5, 25, 50, and 100 μg/mL. The standards and samples were transferred to 96-well plates, and their absorbance was measured at 405 nm. The melanin-associated concentration was calculated from the standard curve and expressed as μg/mL.

### 2.15 Establishment of the vitiligo mouse model and evaluation of therapeutic efficacy

A vitiligo-like depigmentation model was established using 4-week-old female C57BL/6 mice. After shaving the dorsal hair, 40% monobenzone cream was topically applied once daily for 2 consecutive weeks. Mice exhibiting >50% mottled depigmentation on the dorsal skin were randomly assigned to six groups: Model, Gel//HA, Gel@bFGF/TYR//HA, Gel//HA@MPDA, Gel@bFGF/TYR//HA@MPDA, and a tacrolimus-positive control group. The Gel//HA group served as the blank microneedle control and consisted of the same dual-layer GelMA/HAMA//HA matrix without MPDA, bFGF, or TYR, thereby controlling for the effects of microneedle insertion and the matrix materials. The microneedles were applied once every 3 days. This dosing interval was determined by the overall delivery characteristics of the dual-layer system, particularly the rapid dissolution of the HA compartment and the corresponding early release of MPDA, which required periodic replenishment during treatment. Two microneedle patches were applied symmetrically along the dorsal midline, inserted into the depigmented area, and secured with medical adhesive tape. Depigmentation was evaluated according to the method previously reported by our group [29], which was adapted from Zhu et al. [35]. The percentage of the visibly depigmented area within the predefined exposed dorsal skin region was estimated, and the severity was scored as follows: 0 points, no depigmentation (0%); 1 point, >0–5%; 2 points, >5–25%; 3 points, >25–50%; 4 points, >50–75%; and 5 points, >75–100% depigmentation. After 3 weeks of treatment, mice were euthanized, and dorsal skin tissues were collected for histological staining and transcriptomic analysis.

Dorsal skin tissues were fixed in 4% paraformaldehyde, dehydrated through graded ethanol, cleared in xylene, embedded in paraffin, and sectioned at a thickness of 5 μm. H&E and Masson–Fontana staining were performed according to standard protocols to evaluate tissue morphology and melanin deposition, respectively. The melanin-positive area was quantified using ImageJ software. Immunofluorescence staining was performed using primary antibodies against CD8, IFN-γ, TNF-α, SOD2, CAT, GPX4, and 4-HNE, followed by the corresponding fluorescent secondary antibodies and DAPI staining. Tissue ROS levels were detected in frozen skin sections using a dihydroethidium (DHE) probe. Fluorescence signals were quantified as integrated density using ImageJ software. Images for each marker were acquired and analyzed under consistent conditions across all groups.

### 2.16 Transcriptomic analysis

To elucidate the molecular mechanisms of the therapeutic effects, transcriptomic sequencing was performed on dorsal skin tissues from the model group and the Gel@bFGF/TYR//HA@MPDA group (n = 3 per group).

#### 2.16.1 RNA extraction and quality control

Total RNA was extracted from frozen skin tissues using TRIzol® Reagent (Invitrogen, USA) according to the manufacturer's instructions. RNA integrity and concentration were assessed using an Agilent 5300 Bioanalyzer and a NanoDrop 2000 spectrophotometer. Only high-quality RNA samples (OD_260/280_ = 1.8-2.2, *RQN* ≥ 6.5) were used for library construction.

#### 2.16.2 Library preparation and sequencing

The RNA-seq libraries were prepared using the Illumina® Stranded mRNA Prep kit6. Briefly, mRNA was isolated via the polyA selection method using oligo(dT) beads and then fragmented. Double-stranded cDNA was synthesized with random hexamer primers, followed by end-repair, A-tailing, and adapter ligation. After size selection (300-400 bp) and PCR amplification, libraries were sequenced on the Illumina NovaSeq platform in paired-end 150 bp (PE150) mode.

#### 2.16.3 Data processing and read mapping

Raw sequencing data were processed using fastp to remove adapter sequences and low-quality reads10. A total of 39.52 Gb of Clean Data was obtained across six samples, with each sample yielding at least 6.29 Gb and a Q30 base percentage exceeding 96.23%. The high-quality reads were aligned to the *Mus musculus* reference genome (GRCm39, Ensembl) using HISAT2 software. The mapping rates for individual samples ranged from 84.22% to 97.20%.

#### 2.16.4 Differential expression and functional enrichment

Gene expression levels were quantified using RSEM1212. Differentially expressed genes (DEGs) between the Model and treatment groups were identified using DESeq2. The screening criteria were set at: |log_2_(Fold Change)|≥2; P-adj ≤0.05.Functional enrichment analyses, including Gene Ontology (GO) and Kyoto Encyclopedia of Genes and Genomes (KEGG), were performed using Goatools and Python scipy. All bioinformatics analyses were conducted on the Majorbio Cloud Platform (https://cloud.majorbio.com).

### 2.17 Statistical analysis

Data are presented as the mean ± standard deviation (SD) from at least three independent experiments. Comparisons among multiple groups were performed using one-way analysis of variance (ANOVA), while datasets involving both treatment and time were analyzed using two-way ANOVA, followed by an appropriate multiple-comparison test. Statistical significance was defined as *P* < 0.05. Significance levels are indicated as *P* < 0.05, *P < 0.01, and **P < 0.001; n.s. indicates no significant difference.

## 3 Results and discussion

### 3.1 Keratinocyte membrane-coated PDA nanoparticles exhibit enhanced cellular uptake and antioxidant activity

To construct a nanosystem capable of efficient epidermal delivery and alleviation of oxidative stress, PDA nanoparticles were first synthesized through the oxidative self-polymerization of dopamine under alkaline conditions. KC membranes were subsequently isolated from HaCaT cells and coated onto the PDA nanoparticles through coextrusion to obtain KC-membrane-biomimetic MPDA nanoparticles (Fig. 1A–C). PDA possesses a conjugated polycatechol–quinone structure resembling that of natural eumelanin, which provides abundant redox-active groups for scavenging ROS. Meanwhile, KCs are an important component of the epidermal microenvironment and participate in melanosome uptake, pigment distribution, and KC–melanocyte communication. Therefore, KC membrane coating was introduced to improve the interaction of PDA nanoparticles with epidermal KCs while preserving their melanin-like antioxidant properties.

**Fig. 1:**
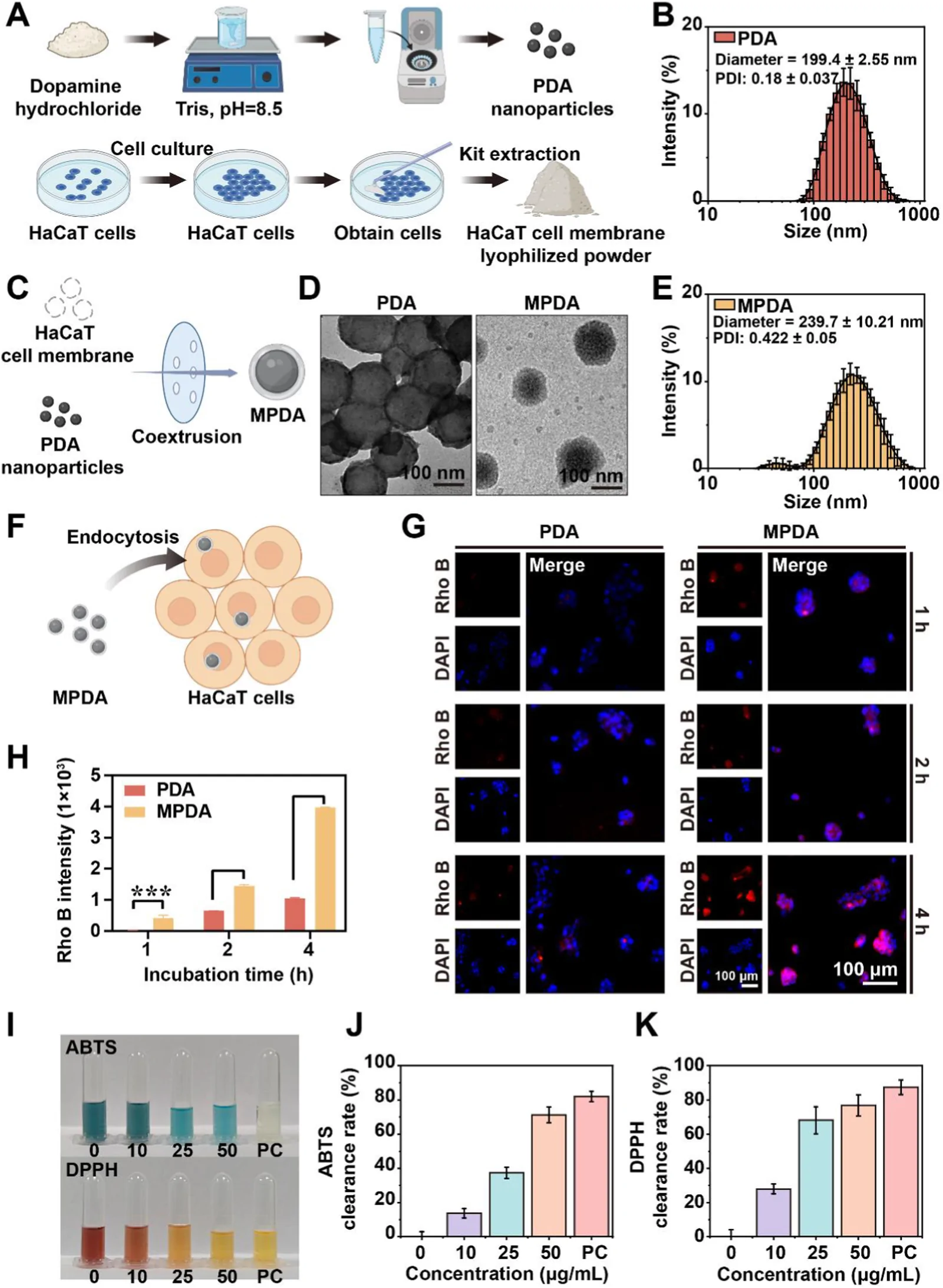
Preparation, characterization, cellular uptake, and antioxidant activity of biomimetic melanin nanoparticles. (A) Schematic illustration of the preparation of PDA nanoparticles and isolation of keratinocyte (KC) membranes. (B) DLS size distribution and corresponding polydispersity index (PDI) of PDA nanoparticles. (C) Schematic illustration of MPDA preparation by coating PDA nanoparticles with KC membranes. (D) TEM images of PDA and MPDA nanoparticles. (E) DLS size distribution and corresponding PDI of MPDA nanoparticles. (F) Schematic illustration of MPDA uptake by KCs. (G) Representative fluorescence images of HaCaT cells incubated with Rho B-labeled PDA or MPDA for 1, 2, and 4 h. (H) Flow cytometric quantification of the mean intracellular Rho B fluorescence intensity in HaCaT cells after incubation with PDA or MPDA for the indicated periods. (I) Representative photographs showing the color changes in the ABTS and DPPH radical-scavenging assays at PDA concentrations of 0, 10, 25, and 50 μg/mL. Trolox and vitamin C were used as the positive controls for the ABTS and DPPH assays, respectively. Quantitative analysis of the (J) ABTS and (K) DPPH radical-scavenging efficiencies of PDA at different concentrations.

DLS analysis showed that the PDA nanoparticles had an average hydrodynamic diameter of 199.4 ± 2.55 nm and a PDI of 0.18 ± 0.037 (Fig. 1B). TEM images revealed that the PDA nanoparticles exhibited a relatively uniform morphology, whereas MPDA displayed a distinct membrane-like layer surrounding the PDA core (Fig. 1D). Following KC membrane coating, the average hydrodynamic diameter increased to 239.7 ± 10.21 nm, with a PDI of 0.422 ± 0.05 (Fig. 1E). The increase in particle size, together with the membrane-like structure observed by TEM, supported the successful coating of PDA nanoparticles with KC membranes. Although the PDI increased after membrane coating, the MPDA nanoparticles remained within a size range suitable for subsequent incorporation into the microneedle system.

To determine whether KC membrane coating enhanced nanoparticle uptake by KCs, PDA and MPDA were labeled with Rho B and incubated with HaCaT cells for 1, 2, and 4 h (Fig. 1F). Fluorescence microscopy showed a time-dependent increase in intracellular red fluorescence for both nanoparticles, with consistently stronger signals observed in the MPDA-treated cells (Fig. 1G). Flow cytometric analysis further showed that the mean intracellular Rho B fluorescence intensity of MPDA was significantly higher than that of PDA at each time point (Fig. 1H). These results demonstrate that KC membrane coating enhanced the cellular uptake of PDA nanoparticles by HaCaT cells. The enhanced cellular uptake may be associated with the retention of membrane proteins involved in cell adhesion and intercellular communication on the KC membrane surface. These membrane components may facilitate nanoparticle attachment and internalization through homotypic membrane interactions and adhesion-enhanced endocytosis. In addition, cell membrane cloaking has been reported to reduce nonspecific protein adsorption and improve the biological interactions of nanoparticles [24], [25]. The PDA core also contains phenolic hydroxyl, amine, and quinone groups capable of forming hydrogen bonds and π–π interactions with cell-surface molecules [36]. Collectively, the KC membrane and PDA core may establish multiple interactions with the cell surface, thereby promoting the intracellular delivery of MPDA. Nevertheless, because the specific uptake pathways and membrane proteins involved were not directly examined in the present study, the precise mechanism underlying the enhanced internalization requires further investigation.

Given that excessive ROS accumulation is a key pathological feature of vitiligo, the radical-scavenging activity of PDA was subsequently evaluated using ABTS and DPPH assays. As the PDA concentration increased from 10 to 50 μg/mL, the characteristic colors of both ABTS and DPPH solutions gradually faded, indicating concentration-dependent radical scavenging (Fig. 1I). Quantitative analysis confirmed that the ABTS radical-scavenging efficiency increased progressively with increasing PDA concentration and reached 71.2% at 50 μg/mL (Fig. 1J). A similar concentration-dependent trend was observed in the DPPH assay, in which PDA achieved a scavenging efficiency of 76.8% at 50 μg/mL (Fig. 1K). Although its radical-scavenging activity remained lower than that of the corresponding positive controls, Trolox and vitamin C, these results confirmed the intrinsic and dose-dependent antioxidant capacity of PDA. This activity can be attributed to the abundant catechol and quinone groups within its melanin-like structure, which can donate electrons or hydrogen atoms to neutralize free radicals [36].

Taken together, the particle-size changes, membrane-like morphology, enhanced cellular internalization, and concentration-dependent radical-scavenging activity demonstrate the successful construction and principal functional properties of MPDA. These characteristics support its subsequent incorporation into the bilayer microneedle system for localized epidermal delivery and modulation of the oxidative microenvironment in vitiligo lesions.

### 3.2 Physicochemical characterization and mechanical optimization of GelMA/HAMA hydrogels

Because free nanoparticles have limited ability to penetrate the intact stratum corneum, hydrogel microneedles were employed to facilitate the localized delivery of MPDA into epidermal tissues [30], [37]. The hydrogel matrix must provide sufficient mechanical strength for skin penetration while retaining the loaded agents for subsequent release. To meet these requirements, gelatin and HA were modified with methacrylic anhydride to obtain the photocrosslinkable derivatives GelMA and HAMA, respectively. By varying the HAMA content, the network structure and mechanical properties of the resulting GelMA/HAMA hydrogels could be adjusted to meet the requirements of microneedle fabrication.

Successful methacrylation of gelatin and HA was confirmed by ^1^H NMR spectroscopy. Compared with unmodified gelatin, GelMA exhibited characteristic vinyl proton peaks at *δ* = 5.65 and 5.33 ppm. Similarly, HAMA displayed vinyl proton signals at *δ* = 6.10 and 5.71 ppm, together with a methyl proton signal at *δ* = 1.86 ppm (Fig. 2A and B). These characteristic signals confirmed the introduction of methacrylate groups, providing reactive sites for subsequent photocrosslinking. Rheological measurements further demonstrated the formation of stable hydrogel networks. Following photocrosslinking, the storage modulus (G′) remained higher than the loss modulus (G″) in all formulations during the time sweep (Fig. 2C). Moreover, the frequency-sweep profiles showed a progressive increase in G′ as the HAMA content increased from 2% to 10% (Fig. 2D), indicating that the stiffness of the hydrogel network could be effectively regulated by adjusting its composition.

**Fig. 2:**
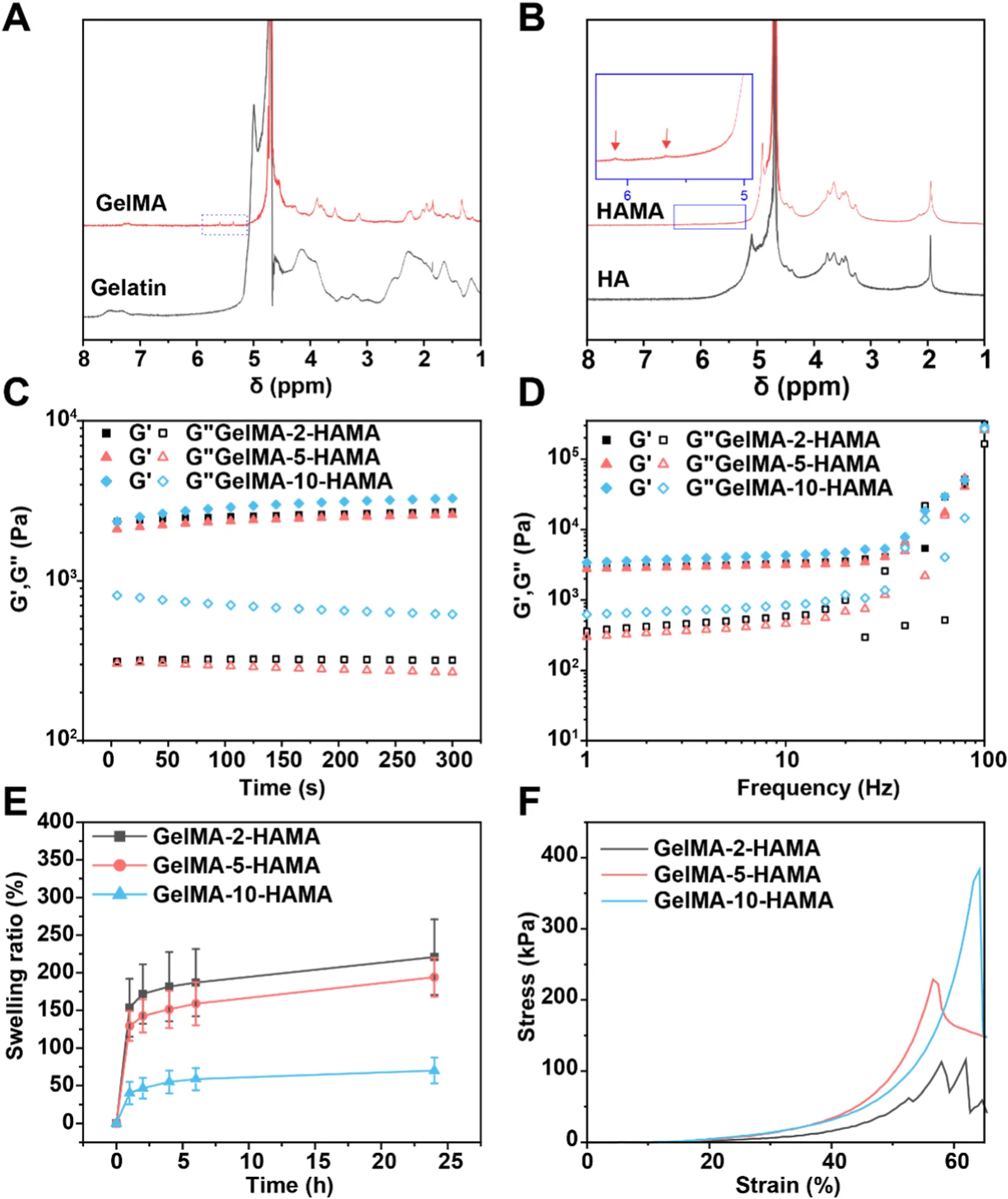
Preparation and characterization of hydrogel materials. (A) ^1^H NMR spectra of gelatin and GelMA. (B) ^1^H NMR spectra of HA and HAMA. (C) Time-sweep rheological profile of the hydrogel. (D) Frequency sweep rheological profile of the hydrogel. (E) Swelling ratio curve of the hydrogel. (F) Compressive stress–strain curves of the hydrogel.

Changes in HAMA content also influenced the hydration behavior of the hydrogels. The formulation containing 2% HAMA exhibited the highest swelling ratio of 220.8 ± 50.3%, whereas increasing the HAMA content to 10% reduced the swelling ratio to 70.1 ± 17.2% (Fig. 2E). This decrease was likely attributable to the formation of a denser crosslinked network, which restricted water penetration and polymer-chain expansion. Such composition-dependent swelling behavior may influence the diffusion and retention of encapsulated agents within the hydrogel matrix, providing a basis for regulating their subsequent release.

The mechanical properties of the hydrogels showed a similar dependence on HAMA content. As the proportion of HAMA increased, the hydrogels exhibited greater resistance to compressive deformation. The formulation containing 10% HAMA achieved the highest compressive modulus of 383.4 kPa and also showed a greater fracture strain (Fig. 2F). This combination of stiffness and deformability is advantageous for microneedle fabrication because it allows the needles to withstand the force applied during skin insertion while reducing the risk of premature structural failure. Taken together, these results demonstrate that the physicochemical and mechanical properties of GelMA/HAMA hydrogels can be readily regulated by varying the HAMA content. Based on its superior mechanical performance, the formulation containing 10% HAMA was selected for the subsequent fabrication of bilayer microneedles.

### 3.3 Fabrication, skin penetration, and compartment-dependent release of bilayer hydrogel microneedles

To achieve efficient skin penetration and temporally differentiated delivery of MPDA, bFGF, and TYR, a bilayer hydrogel microneedle system was fabricated through sequential casting and photocrosslinking (Fig. S1). The crosslinked GelMA/HAMA layer formed the outer portion of the microneedles and was loaded with bFGF and TYR, whereas the non-crosslinked HA compartment contained MPDA. This configuration combined the mechanical support provided by the GelMA/HAMA network with the rapid hydration and dissolution characteristics of HA, thereby allowing the three therapeutic components to be incorporated into distinct compartments according to their intended release profiles.

Fluorescence imaging clearly distinguished the Rho B-labeled HA compartment from the FITC-labeled GelMA/HAMA layer in both the top and side views of the microneedle arrays (Fig. 3A). SEM further revealed uniformly shaped pyramidal microneedles with sharp tips and smooth sidewalls (Fig. 3B), indicating good mold fidelity and polymer curing. The fabricated microneedles exhibited a height of approximately 800 μm, sufficient to traverse the stratum corneum. The continuous interface between the two fluorescent regions confirmed the successful formation of the bilayer structure through sequential casting. Mechanical testing further showed that increasing the HAMA content improved the resistance of individual microneedles to compressive deformation (Fig. 3C and D). Among the tested formulations, GelMA-10-HAMA microneedles exhibited the highest fracture force, reaching 0.234 ± 0.014 N per needle. This value exceeded the force generally required for skin penetration, approximately 0.1–0.2 N per needle [38], indicating that the optimized microneedles possessed sufficient mechanical strength for insertion without premature deformation or fracture.

**Fig. 3:**
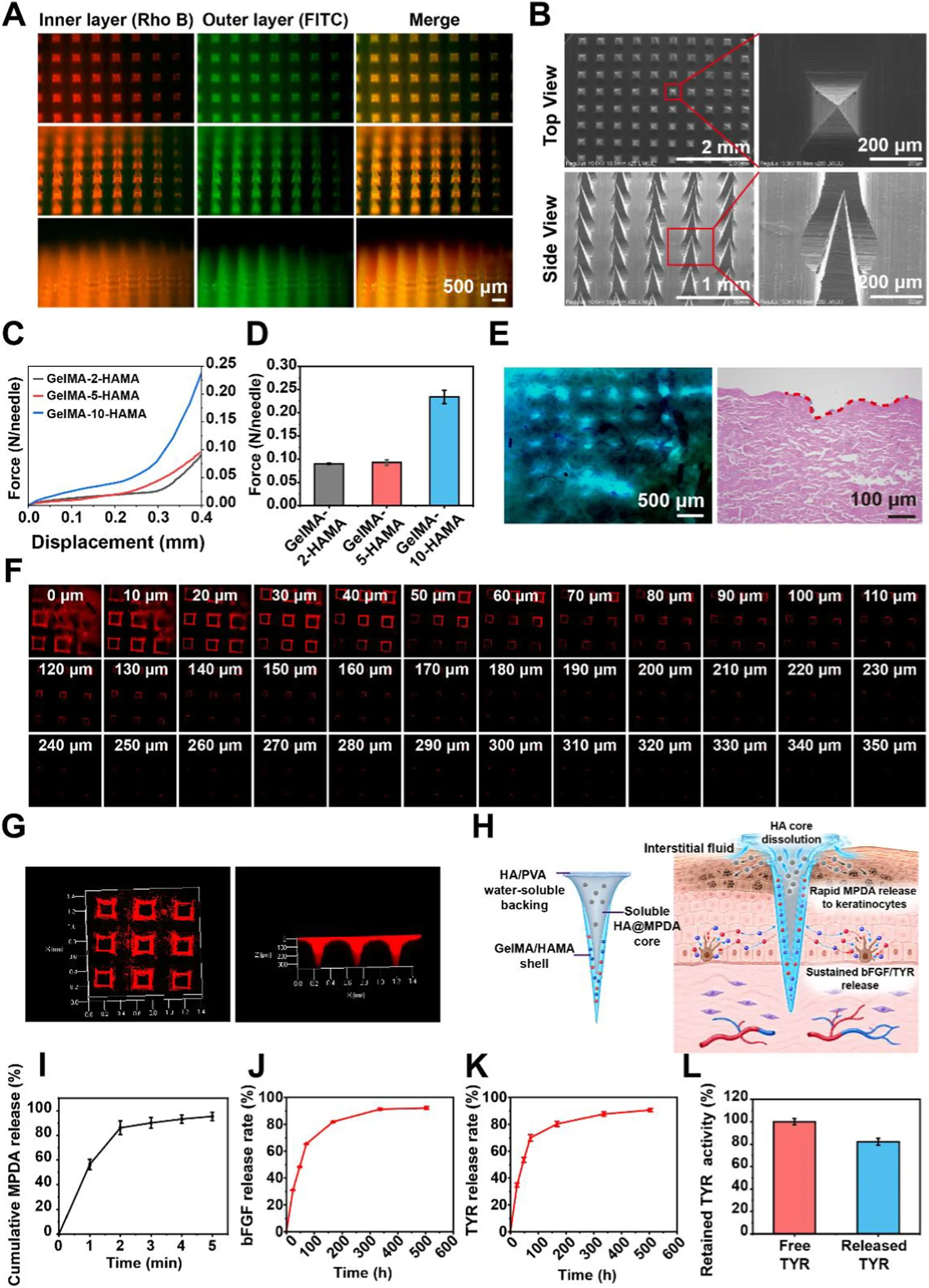
Fabrication, characterization, skin penetration, and release performance of double-layer hydrogel microneedles. (A) Top- and side-view fluorescence images of the double-layer microneedles, with the inner HA layer labeled with Rho B and the outer GelMA/HAMA layer labeled with FITC. (B) SEM images of the double-layer hydrogel microneedles. (C) Single-needle compression curves of GelMA/HAMA microneedles containing different HAMA mass fractions. (D) Quantitative analysis of the mechanical strength of GelMA/HAMA microneedles containing different HAMA mass fractions. (E) Methylene blue staining of the insertion sites and H&E staining of porcine skin after microneedle insertion. (F) Confocal z-stack images and (G) three-dimensional confocal reconstruction of the microneedles inserted into porcine skin. (H) Schematic illustration of the double-layer microneedle structure, intradermal dissolution, and differential release of MPDA, bFGF, and TYR. (I) Cumulative release profile of MPDA from the HA compartment of the bilayer microneedles. Cumulative release profiles of (J) bFGF and (K) TYR from bulk GelMA/HAMA hydrogels prepared with the same composition and photocrosslinking conditions as the outer microneedle layer. (L) Retained enzymatic activity of TYR released from the GelMA/HAMA hydrogel at 72 h relative to that of free TYR at the same concentration. Blank GelMA/HAMA release medium was used as the background control.

The skin-penetration performance of the optimized microneedles was subsequently evaluated using porcine skin. Methylene blue staining revealed regularly distributed insertion sites corresponding to the microneedle array, while H&E staining showed well-defined microchannels extending across the stratum corneum and into the underlying skin (Fig. 3E). Confocal z-stack imaging and three-dimensional reconstruction further demonstrated that the fluorescent microneedles penetrated to a depth of approximately 350 μm (Fig. 3F and G). Together, these results confirmed that the bilayer microneedles could effectively overcome the stratum corneum and establish continuous pathways for intradermal delivery.

The distinct physicochemical properties of the two hydrogel compartments resulted in different release behaviors after insertion (Fig. 3H). The crosslinked GelMA/HAMA outer layer first contacted the skin and provided the mechanical support required to create microchannels. Interstitial fluid entering through these channels subsequently reached the non-crosslinked HA compartment, leading to its rapid hydration and dissolution. MPDA could therefore be released into the fluid-filled microchannels and become accessible to KCs within the viable epidermis. By contrast, the crosslinked GelMA/HAMA network remained in the tissue for a longer period and allowed the comparatively slower release of bFGF and TYR. Thus, the differential release profiles were primarily determined by the distinct crosslinking states and dissolution properties of the two compartments, rather than solely by their spatial positions within the microneedles.

The loading amounts of bFGF and TYR in the GelMA/HAMA compartment were 65.62 ± 0.25 ng and 63.57 ± 0.01 μg per microneedle patch, respectively. MPDA was rapidly released from the non-crosslinked HA compartment of the bilayer microneedles (Fig. 3I). TEM and DLS analyses showed that the released MPDA retained a morphology and particle-size distribution comparable to those before incorporation into the microneedles (Fig. S2), suggesting that the fabrication and release processes did not markedly alter its structural characteristics. By comparison, bFGF and TYR exhibited biphasic release from bulk GelMA/HAMA hydrogels prepared using the same composition and crosslinking conditions as the outer microneedle layer (Fig. 3J and K). Approximately 70% of both agents was released within the first 72 h, followed by a slower release phase extending to 21 days. TYR released from the GelMA/HAMA hydrogel at 72 h retained approximately 82.2% of the activity of free TYR at the same concentration (Fig. 3L), indicating that the encapsulation, photocrosslinking, and release processes largely preserved its catalytic activity. These results support the intended compartment-dependent delivery, characterized by rapid MPDA release from the HA compartment and more sustained release of bFGF and TYR from the crosslinked GelMA/HAMA network.

### 3.4 Effects of bilayer hydrogel microneedles on HaCaT cell proliferation, migration, and oxidative stress responses

Before evaluating the cellular effects of the bilayer microneedles, the cytocompatibility of the individual therapeutic components was examined. MPDA did not markedly affect HaCaT cell viability at concentrations of 20–100 μg/mL, whereas a clear decrease was observed when the concentration was increased to 200 and 400 μg/mL (Fig. S3A). bFGF showed no detectable cytotoxicity over the tested concentration range of 1–200 ng/mL (Fig. S3B). TYR was also well tolerated by HaCaT cells at relatively low concentrations, although a moderate reduction in cell viability occurred at 100–200 U/mL (Fig. S3C). These results defined suitable concentration ranges for subsequent experiments and indicated that the three functional components were cytocompatible at their intended working concentrations.

The effects of the complete microneedle formulations on HaCaT cell growth were then evaluated using microneedle extracts. No apparent differences in cell viability were observed among the groups after 1 day of incubation. By day 3, however, the Gel@bFGF/TYR//HA@MPDA group exhibited significantly higher cell viability than the other groups (Fig. 4A). Ki67 immunofluorescence staining showed a similar time-dependent pattern. Only limited Ki67 fluorescence was observed on day 1, whereas its expression increased markedly by day 3, particularly in cells treated with Gel@bFGF/TYR//HA@MPDA extracts (Fig. 4B). Quantitative analysis confirmed that the composite formulation produced the highest Ki67 fluorescence intensity among the tested groups (Fig. 4C). These findings suggest that the complete formulation provided a more favorable environment for HaCaT cell proliferation than either the blank microneedles or the partially loaded formulations.

**Fig. 4:**
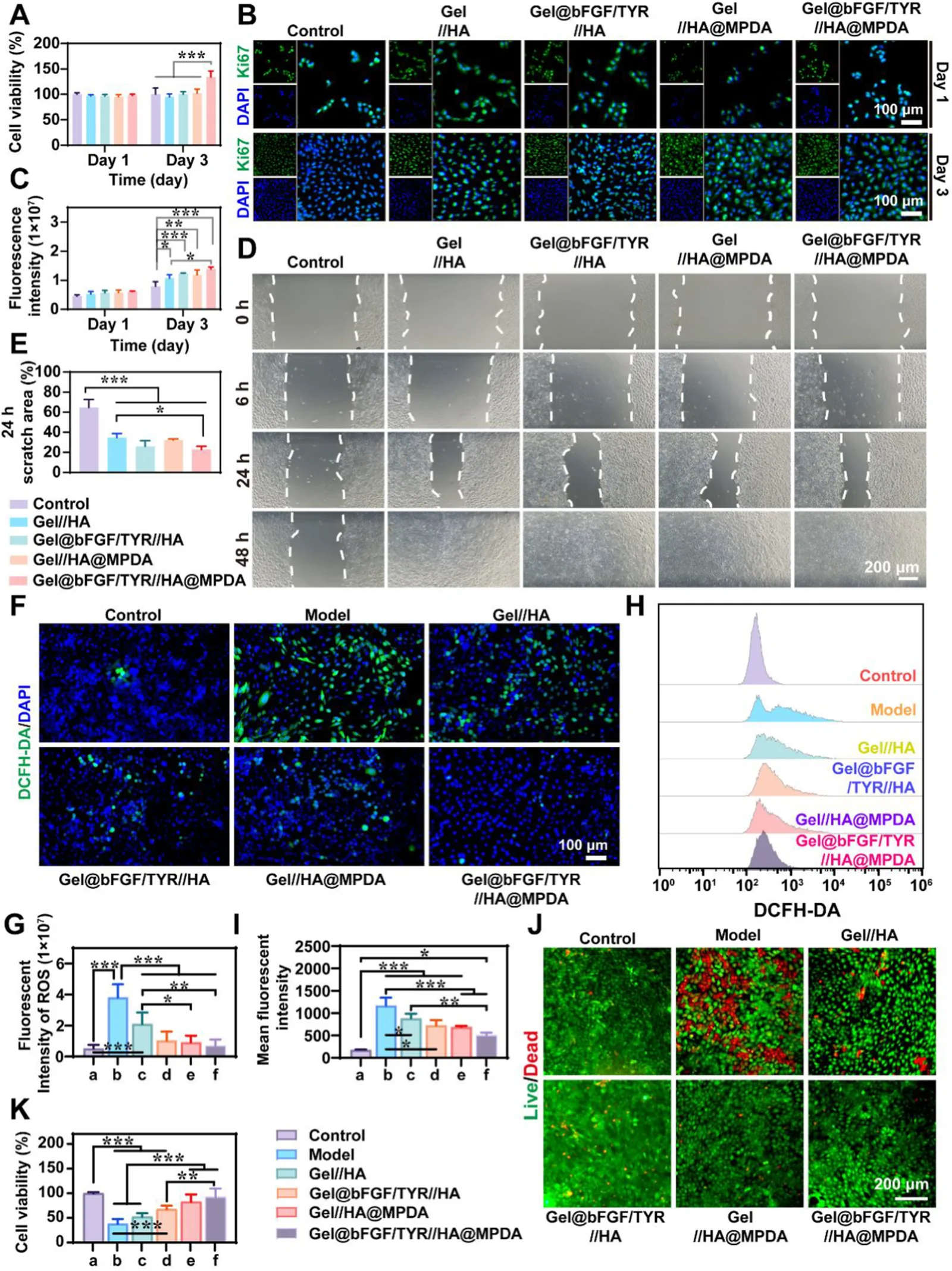
Effects of hydrogel microneedles on HaCaT cell proliferation, migration, and protection against oxidative injury. (A) Viability of HaCaT cells after treatment with extracts from different hydrogel microneedle formulations for 1 and 3 days. (B) Representative Ki67 immunofluorescence images of HaCaT cells treated with different hydrogel microneedle extracts and (C) corresponding quantitative analysis of Ki67 fluorescence intensity. (D) Scratch assay of HaCaT cells treated with different hydrogel microneedle extracts and (E) quantitative analysis of the residual scratch area after 24 h. (F) Representative DCFH-DA fluorescence images of intracellular ROS and (G) corresponding quantitative analysis of fluorescence intensity in H_2_O_2_-injured HaCaT cells after treatment with different hydrogel microneedle extracts for 24 h. (H) Representative flow cytometric profiles of DCFH-DA fluorescence in HaCaT cells and (I) corresponding quantitative analysis of intracellular ROS levels. (J) Live/dead fluorescence staining of HaCaT cells following H_2_O_2_-induced injury and subsequent treatment with different hydrogel microneedle extracts for 24 h. (K) CCK-8 analysis of cell viability in untreated normal HaCaT cells and H_2_O_2_-injured HaCaT cells subsequently cultured with or without different hydrogel microneedle extracts.

Because KC migration contributes to epidermal repair and the re-establishment of a functional microenvironment for melanocytes, a scratch assay was performed to examine the effects of the microneedle extracts on HaCaT cell migration. The untreated control group showed relatively slow closure of the scratched area, whereas all microneedle extract-treated groups exhibited more rapid narrowing of the wound gap (Fig. 4D). At 24 h, the residual scratch area was lower in the drug-loaded groups than in the control group, with the Gel@bFGF/TYR//HA@MPDA group showing the smallest remaining scratch area (Fig. 4E). The differences among the loaded formulations were less pronounced at later time points as the scratches gradually approached closure. Together with the proliferation results, these findings indicate that the combined formulation promoted HaCaT cell growth and migration, both of which may support epidermal recovery.

KCs in vitiligo lesions are exposed to persistent oxidative stress and exhibit impaired antioxidant defenses, disrupted cellular homeostasis, and increased susceptibility to injury [1], [14], [17]. To reproduce this oxidative environment *in vitro*, HaCaT cells were treated with increasing concentrations of H_2_O_2_. Cell viability remained relatively stable at 200–1200 μmol/L but declined markedly at higher concentrations. Treatment with 1600 μmol/L H_2_O_2_ reduced cell viability to approximately 50% and was therefore selected to establish the oxidative injury model (Fig. S4).

DCFH-DA staining showed weak intracellular fluorescence in untreated control cells, whereas H_2_O_2_ exposure caused a pronounced increase in ROS accumulation (Fig. 4F). Treatment with the different microneedle extracts reduced ROS fluorescence to varying degrees. The blank Gel//HA extract produced a moderate reduction, while extracts containing bFGF/TYR or MPDA showed more evident antioxidant effects. The lowest ROS fluorescence was observed in the Gel@bFGF/TYR//HA@MPDA group (Fig. 4F and G). Flow cytometric analysis confirmed these observations, showing a marked rightward shift in DCFH-DA fluorescence after H_2_O_2_ exposure and a progressive reduction following treatment with the different microneedle extracts (Fig. 4H). Quantitative analysis of the mean fluorescence intensity further demonstrated that the complete formulation produced the greatest reduction in intracellular ROS (Fig. 4I).

The protective effects of the microneedles were further evaluated by live/dead staining and CCK-8 analysis. H_2_O_2_-treated cells showed extensive red fluorescence and a marked reduction in the number of viable cells. In comparison, treatment with the drug-loaded microneedle extracts reduced cell death, with the Gel@bFGF/TYR//HA@MPDA group displaying predominantly live cells and only limited red fluorescence (Fig. 4J). Consistently, H_2_O_2_ exposure reduced cell viability to approximately 38%, whereas the blank Gel//HA extract provided a modest protective effect. The bFGF/TYR- and MPDA-containing formulations further improved cell viability, and the complete formulation restored it to a level approaching that of untreated cells (Fig. 4K). These results demonstrate that the protection afforded by the complete microneedle formulation was not limited to a reduction in DCFH-DA fluorescence but was also accompanied by improved cell survival under oxidative stress.

Western blotting was subsequently performed to determine whether the reduction in oxidative injury was accompanied by changes in stress-associated inflammatory protein expression. H_2_O_2_ exposure increased the expression of IFN-γ and TNF-α compared with untreated control cells (Fig. S5A). Treatment with the microneedle extracts reduced IFN-γ expression to varying degrees, with the Gel@bFGF/TYR//HA@MPDA group showing the most pronounced decrease (Fig. S5B). TNF-α expression was also lower in the treatment groups than in the model group, although the differences among the individual microneedle formulations were relatively modest. These findings suggest that alleviation of oxidative stress was accompanied by attenuation of the inflammatory response in injured HaCaT cells.

The protective effects of the complete formulation likely resulted from the complementary functions of its components. The catechol- and quinone-rich structure of MPDA confers intrinsic radical-scavenging capacity [10], [22], [31], [36], [39], whereas bFGF supports cell survival and repair under adverse conditions [40]. TYR was incorporated primarily to support subsequent melanogenic processes and remained cytocompatible within the selected concentration range. Their combined delivery therefore reduced intracellular ROS accumulation, limited cell death, and preserved the proliferative and migratory activities of HaCaT cells. Collectively, these results indicate that Gel@bFGF/TYR//HA@MPDA microneedles can protect KCs against oxidative injury while supporting cellular behaviors relevant to epidermal recovery.

### 3.5 Bilayer hydrogel microneedles alleviate oxidative injury and increase melanin content in B16-F10 cells

B16-F10 cells were used as a melanogenic cell model to examine whether the antioxidant effects observed in HaCaT cells could also protect pigment-producing cells under oxidative stress. Although B16-F10 cells are derived from murine melanoma and cannot fully reproduce the physiology of primary human melanocytes, they retain active melanogenic machinery and produce readily quantifiable melanin. They have therefore been widely used to evaluate melanogenic activity and its associated signaling pathways [41], [42]. In the absence of oxidative injury, treatment with bFGF/TYR, MPDA, or their combination did not reduce B16-F10 cell viability during the 3-day culture period (Fig. S6A). The viability of all groups remained close to or above 100%, with the combined MPDA/bFGF/TYR group showing a modest increase on day 3. These results excluded overt cytotoxicity as a confounding factor in the subsequent experiments.

The individual components also produced distinguishable effects on cellular melanin content. After 24 h, the measured melanin concentration increased from 3.7 μg/mL in the control group to 8.1 μg/mL following either bFGF/TYR or MPDA treatment. Combining MPDA with bFGF/TYR further increased the melanin-associated content to 13.5 μg/mL, which was significantly higher than that achieved by either treatment alone (Fig. S6B). The increase induced by bFGF/TYR is consistent with the involvement of TYR in melanin formation and the supportive effect of bFGF on cellular activity. In the MPDA-containing groups, however, the measured value may include both intracellular MPDA and endogenously synthesized melanin. The assay therefore reflects total cellular melanin-associated content rather than endogenous melanin synthesis alone.

The antioxidant effects of the microneedle formulations were subsequently evaluated in H_2_O_2_-injured B16-F10 cells. DCFH-DA imaging showed that H_2_O_2_ exposure increased ROS fluorescence by 71-fold relative to untreated control cells (Fig. 5A and B). Compared with the model group, Gel//HA treatment reduced the fluorescence intensity to approximately 0.4-fold, indicating a partial protective effect of the hydrogel matrix. Gel@bFGF/TYR//HA further decreased the ROS signal to approximately 0.17-fold of that in the model group. More pronounced reductions were observed in the MPDA-containing groups, in which the fluorescence intensity fell to less than 0.06-fold of the model level. Among all formulations, Gel@bFGF/TYR//HA@MPDA produced the lowest residual ROS signal, approaching the level observed in untreated cells. These results indicate that MPDA made a major contribution to intracellular ROS elimination, while the complete formulation achieved the strongest overall antioxidant effect.

**Fig. 5:**
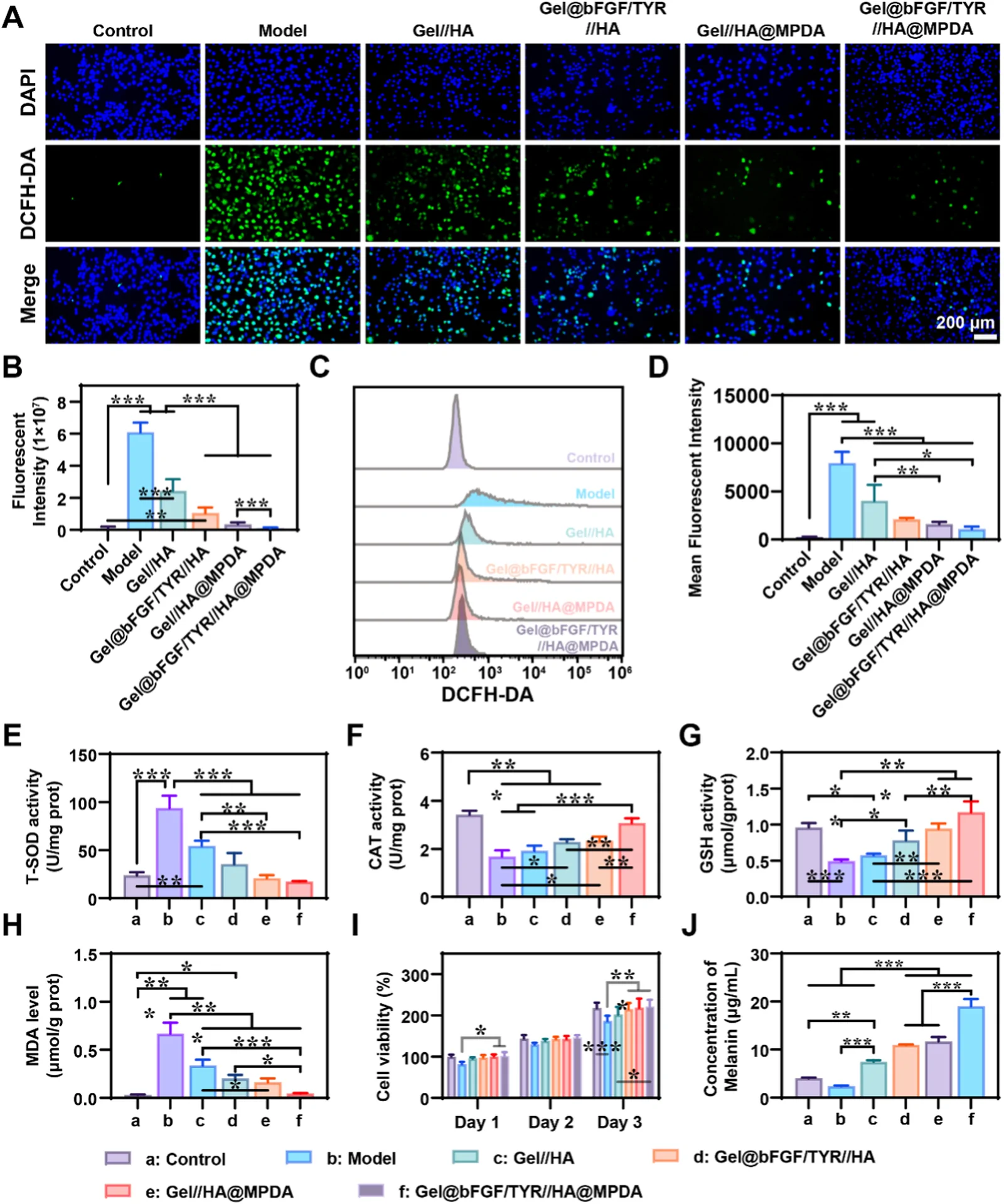
Protective effects of hydrogel microneedles against H_2_O_2_-induced oxidative injury in B16-F10 cells. (A) Representative DCFH-DA fluorescence images of H_2_O_2_-injured B16-F10 cells after treatment with different hydrogel microneedle extracts and (B) corresponding quantitative analysis of fluorescence intensity. (C) Representative flow cytometric profiles of DCFH-DA fluorescence and (D) corresponding quantitative analysis of intracellular ROS levels. Measurements of (E) total superoxide dismutase (T-SOD) activity, (F) catalase (CAT) activity, (G) glutathione (GSH) levels, and (H) malondialdehyde (MDA) levels in H_2_O_2_-injured B16-F10 cells after treatment with different hydrogel microneedle extracts. (I) Viability and (J) melanin content of H_2_O_2_-injured B16-F10 cells following treatment with different hydrogel microneedle extracts.

Flow cytometry independently confirmed this trend (Fig. 5C and D). H_2_O_2_ exposure increased the mean DCFH-DA fluorescence intensity by 29-fold relative to untreated control cells. Compared with the model group, Gel//HA treatment reduced the fluorescence intensity to 0.51-fold, whereas Gel@bFGF/TYR//HA and Gel//HA@MPDA reduced it to 0.27- and 0.20-fold, respectively. The complete Gel@bFGF/TYR//HA@MPDA formulation produced the greatest reduction, lowering the mean fluorescence intensity to less than 0.14-fold of the model level. These results further demonstrate that the MPDA-containing formulations exerted a stronger ROS-reducing effect, with the complete formulation providing the greatest overall protection against oxidative stress.

Changes in intracellular antioxidant and oxidative damage markers were consistent with the ROS measurements. H_2_O_2_ exposure increased T-SOD activity from 24 to 94 U/mg protein (Fig. 5E). Given the simultaneous increase in ROS and MDA, this elevation most likely represented a compensatory response to acute oxidative stress rather than an improvement in cellular redox status. T-SOD activity decreased to 55, 36, 21, and 17 U/mg protein following treatment with Gel//HA, Gel@bFGF/TYR//HA, Gel//HA@MPDA, and Gel@bFGF/TYR//HA@MPDA, respectively. The return of T-SOD activity toward the basal level paralleled the reduction in intracellular ROS.

By contrast, CAT activity decreased from 3.4 U/mg protein in untreated cells to 1.7 U/mg protein in the model group (Fig. 5F). The complete formulation restored CAT activity to 3.1 U/mg protein, close to the control level, whereas the other formulations produced only partial recovery. A similar pattern was observed for GSH, which decreased from approximately 0.96 to 0.5 μmol/g protein after H_2_O_2_ exposure (Fig. 5G). Gel//HA and Gel@bFGF/TYR//HA restored GSH to 0.58 and 0.78 μmol/g protein, respectively, while Gel//HA@MPDA restored it to approximately the control level. The complete formulation increased GSH to approximately 1.17 μmol/g protein, exceeding the level detected in untreated cells.

H_2_O_2_-induced oxidative stress was also accompanied by a substantial increase in MDA, from 0.031 μmol/g protein in the control group to approximately 0.668 μmol/g protein in the model group (Fig. 5H). Gel//HA reduced MDA to 0.337 μmol/g protein, whereas Gel@bFGF/TYR//HA and Gel//HA@MPDA further reduced it to 0.204 and 0.163 μmol/g protein, respectively. Gel@bFGF/TYR//HA@MPDA produced the greatest reduction, lowering MDA to a level close to that of untreated cells. These results show that the complete formulation not only reduced overall ROS accumulation but also preserved CAT/GSH-associated antioxidant defenses and limited lipid peroxidation. Nevertheless, the reduction in MDA should be interpreted as evidence of attenuated oxidative membrane damage rather than direct confirmation of ferroptosis inhibition.

The improvement in redox status was accompanied by recovery of cell viability (Fig. 5I). H_2_O_2_ exposure initially reduced B16-F10 cell viability, whereas all microneedle extract-treated groups showed progressive recovery during the following 3 days. On day 3, the Gel@bFGF/TYR//HA@MPDA group exhibited the highest viability among the H_2_O_2_-treated groups, indicating that the combined reduction in oxidative burden and restoration of antioxidant capacity translated into improved cellular survival and proliferation.

Oxidative injury also reduced the measured melanin concentration from 4.07 μg/mL in untreated cells to 2.39 μg/mL in the model group (Fig. 5J). Treatment with Gel//HA increased this value to 7.45 μg/mL, while Gel@bFGF/TYR//HA and Gel//HA@MPDA increased it to nearly 11 μg/mL. The highest melanin-associated content, approximately 19 μg/mL, was observed in the Gel@bFGF/TYR//HA@MPDA group, representing an approximately sevenfold increase relative to the H_2_O_2_-treated model group. The higher value obtained with the complete formulation than with either partially loaded formulation supports a complementary contribution from MPDA-mediated antioxidant protection and bFGF/TYR-associated pigmentation support. However, because MPDA has melanin-like optical properties, the assay cannot distinguish internalized MPDA from newly synthesized melanin. Accordingly, these data demonstrate increased total melanin-associated content but do not independently establish enhanced endogenous melanogenesis.

Taken together, the comparisons among the partially loaded and complete formulations reveal complementary roles of the incorporated components. MPDA-containing microneedles produced the greatest reductions in intracellular ROS and MDA and more effectively restored GSH levels, identifying antioxidant protection as a major contribution of MPDA. The addition of bFGF and TYR further improved cellular recovery and increased melanin-associated content. Consequently, the complete Gel@bFGF/TYR//HA@MPDA formulation brought multiple redox indicators close to their basal levels while producing the strongest pigmentation-associated response. These findings support a coordinated therapeutic effect in which MPDA alleviates the oxidative pressure imposed on B16-F10 cells, while bFGF and TYR provide additional support for cell recovery and pigment formation.

### 3.6 Gel@bFGF/TYR//HA@MPDA microneedles promote repigmentation and attenuate local immune activation in vitiligo-like lesions

A vitiligo-like depigmentation model was established by topical application of 40% monobenzone for two consecutive weeks. Before treatment, all model groups exhibited extensive dorsal depigmentation and comparable Vitiligo Area Scoring Index values, confirming similar disease severity (Fig. 6A and B). During the subsequent 3 weeks, the depigmented area remained largely unchanged in the model group, whereas varying degrees of repigmentation were observed following microneedle treatment. Blank Gel//HA microneedles produced only a moderate improvement, while the bFGF/TYR- and MPDA-containing formulations progressively reduced the lesion area. The most pronounced recovery was observed in the Gel@bFGF/TYR//HA@MPDA group, whose scoring index decreased from 4.7 to 1.3 by week 3. This improvement was also evident from the extensive recovery of skin and hair pigmentation and was greater than that achieved with tacrolimus.

**Fig. 6:**
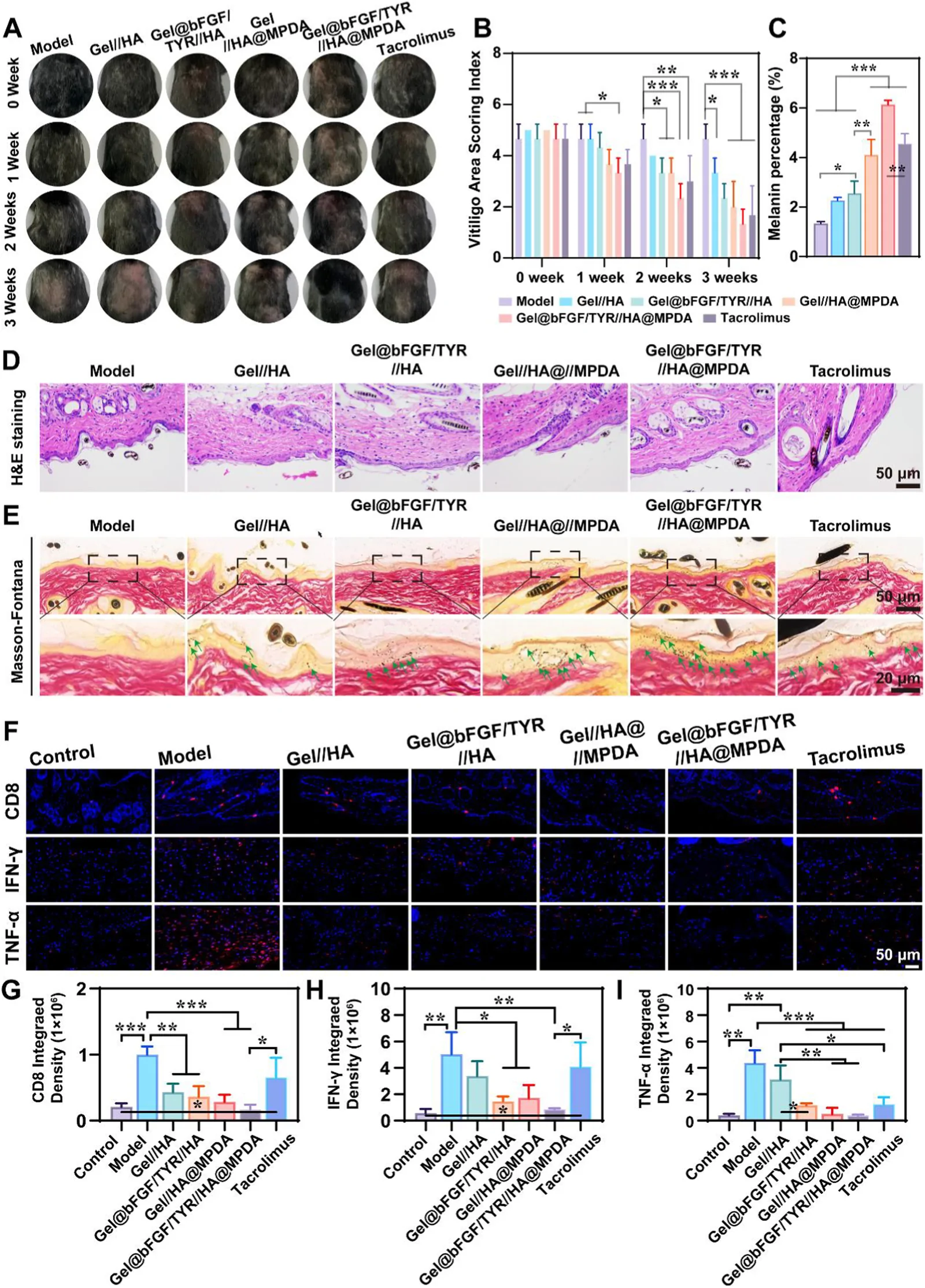
Evaluation of repigmentation and local immune modulation in monobenzone-induced vitiligo-like lesions after microneedle treatment. (A) Representative images of mouse dorsal skin after 3 weeks of treatment. (B) Quantitative scoring of the depigmented lesion areas. (C) Quantitative analysis of epidermal melanin content. (D) Representative H&E-stained skin sections showing epidermal morphology after different treatments. (E) Representative Masson–Fontana-stained sections showing the distribution of melanin granules in skin tissues. (F) Representative immunofluorescence images of CD8, IFN-γ, and TNF-α in skin tissues. Quantitative analysis of the fluorescence intensity of (G) CD8, (H) IFN-γ, and (I) TNF-α.

Histological evaluation was consistent with the macroscopic observations. H&E staining showed that the overall epidermal and dermal architecture remained intact after repeated microneedle application, without obvious treatment-related tissue damage (Fig. 6D). Masson–Fontana staining revealed sparse pigment deposition in the model group, whereas melanin-positive signals gradually increased in the Gel//HA, Gel@bFGF/TYR//HA, and Gel//HA@MPDA groups (Fig. 6E). The complete formulation produced the strongest and most continuous pigment deposition along the epidermis and around hair follicles. Quantitatively, the melanin-positive area in the Gel@bFGF/TYR//HA@MPDA group reached 6.1%, representing an approximately fivefold increase relative to the model group (Fig. 6C).

The greater pigmentation observed in the MPDA-containing groups likely reflects both the delivery of melanin-like MPDA and recovery of the local environment required for pigment maintenance. Meanwhile, bFGF and TYR may provide additional support for cell survival and melanin formation. Because MPDA may itself contribute to pigment-associated staining, however, the increase in Masson–Fontana-positive signals should be interpreted as overall tissue pigment restoration rather than exclusively as *de novo* endogenous melanin synthesis.

Persistent recruitment of cytotoxic CD8^+^ T cells and increased production of IFN-γ and TNF-α are central features of the inflammatory microenvironment in vitiligo [4], [14], [43]. Consistent with this pathological profile, the model group displayed markedly stronger CD8, IFN-γ, and TNF-α fluorescence than normal skin (Fig. 6F). Blank Gel//HA microneedles produced only a partial reduction, whereas the drug-loaded formulations attenuated these immune-related signals to a greater extent. In particular, the complete Gel@bFGF/TYR//HA@MPDA formulation reduced CD8 accumulation to 0.17-fold of the model level and restored it close to that observed in normal skin (Fig. 6G).

IFN-γ and TNF-α showed similar treatment-dependent changes. Both cytokines remained relatively elevated after Gel//HA treatment but were substantially reduced by the bFGF/TYR- and MPDA-containing formulations (Fig. 6H and I). The complete formulation produced the greatest suppression, lowering IFN-γ and TNF-α signals to approximately 0.1 to 0.2-fold of their respective model levels. Its inhibitory effects were also more pronounced than those observed with tacrolimus under the present experimental conditions.

Together, these results show that the superior repigmentation achieved with Gel@bFGF/TYR//HA@MPDA was accompanied by attenuation of CD8^+^ T cell-associated immune activation. The comparisons among formulations further suggest complementary contributions from MPDA-mediated microenvironmental protection and bFGF/TYR-associated support of pigmentation. Although the present data do not establish a direct causal relationship between immune modulation and pigment recovery, they demonstrate that the complete microneedle formulation concurrently improved tissue pigmentation and alleviated the inflammatory state of vitiligo-like lesions.

### 3.7 Gel@bFGF/TYR//HA@MPDA microneedles restore antioxidant defenses and limit lipid peroxidation in vitiligo-like skin

Because persistent oxidative stress contributes to KC dysfunction and melanocyte vulnerability in vitiligo [1], [14], [17], we next examined whether the improvement in pigmentation and local immune status was accompanied by restoration of cutaneous redox homeostasis. ROS-sensitive fluorescence staining showed weak signals in normal skin but extensive ROS accumulation throughout the epidermis and perifollicular regions of the model group (Fig. 7A). Quantitative analysis showed 4.3-fold increase in ROS fluorescence in the model group relative to normal skin. Blank Gel//HA microneedles produced only a partial reduction, whereas the bFGF/TYR- and MPDA-containing formulations decreased ROS accumulation more effectively. The complete Gel@bFGF/TYR//HA@MPDA formulation reduced ROS fluorescence to 0.23-fold of the model level, restoring it close to that of normal skin. In comparison, relatively high ROS signals remained after tacrolimus treatment, indicating that the complete microneedle formulation exerted a stronger local antioxidant effect under the present conditions.

**Fig. 7:**
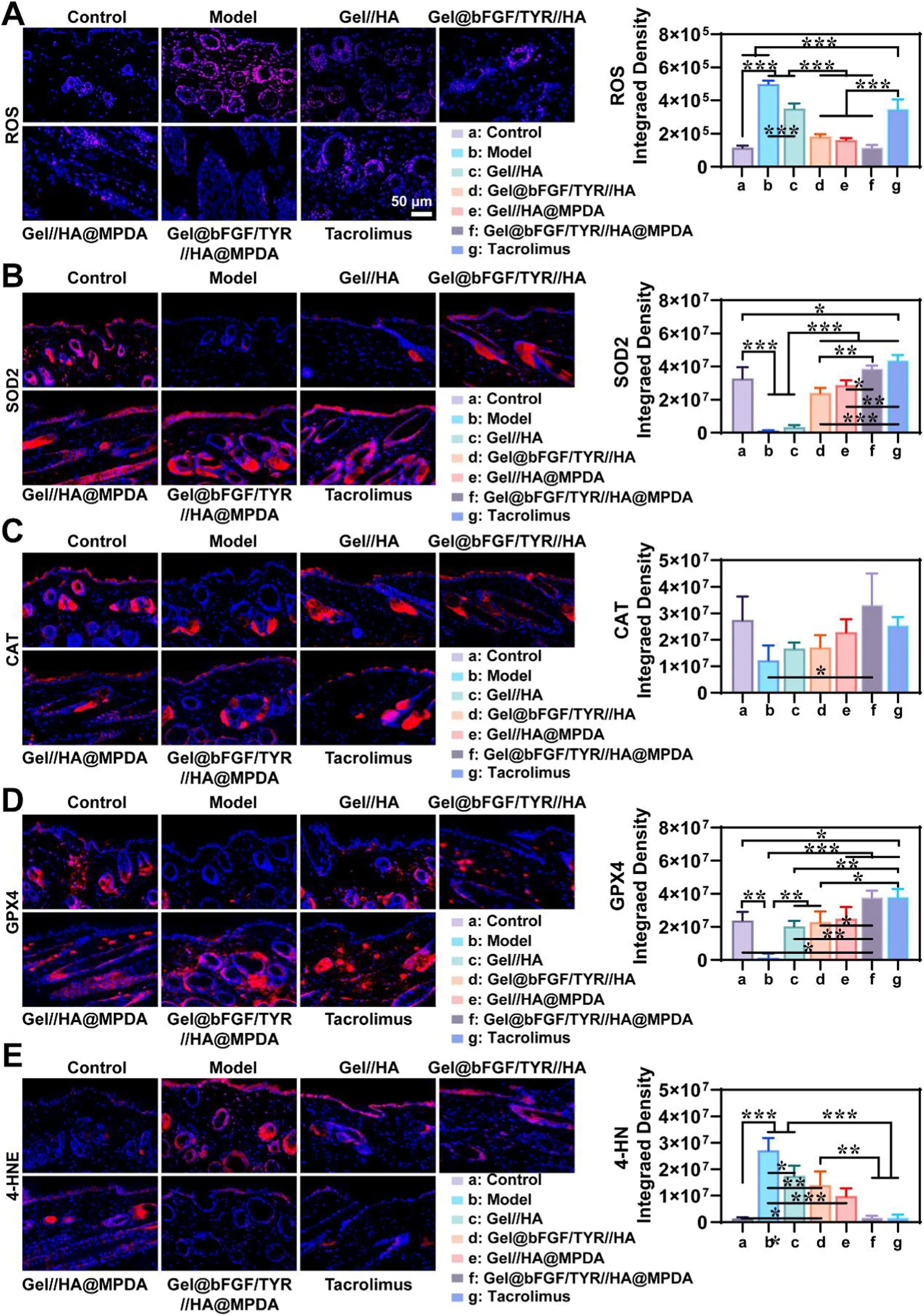
Assessment of oxidative stress, antioxidant defense, and lipid peroxidation in vitiligo-like skin after microneedle treatment. (A) Representative ROS fluorescence images of skin sections and corresponding quantitative analysis of fluorescence intensity. Representative immunofluorescence images and corresponding quantitative analyses of (B) SOD2, (C) CAT, (D) GPX4, and (E) 4-HNE expression in skin tissues after different treatments.

The expression of endogenous antioxidant enzymes was subsequently examined. SOD2 staining was markedly reduced in the model group, reaching 3.5% of the level observed in normal skin (Fig. 7B). Blank Gel//HA treatment resulted in only a limited increase, whereas the drug-loaded microneedles progressively restored SOD2 expression. Both MPDA-containing formulations showed pronounced recovery, and the Gel@bFGF/TYR//HA@MPDA group restored SOD2 expression to a level comparable to or slightly higher than that of normal skin. This result differs from the acute compensatory increase in total SOD activity observed in H_2_O_2_-treated B16-F10 cells, suggesting that prolonged oxidative injury *in vivo* was instead associated with impairment of the tissue antioxidant defense system.

CAT expression showed a similar but less pronounced treatment-dependent trend (Fig. 7C). The model group exhibited lower CAT fluorescence than normal skin, while the different microneedle formulations produced varying degrees of recovery. The complete formulation increased CAT expression to 2.7-fold of the model level and showed the highest mean value among the microneedle-treated groups. GPX4, a key enzyme responsible for reducing phospholipid hydroperoxides, was markedly suppressed in vitiligo-like skin (Fig. 7D). Its fluorescence intensity in the model group was less than 0.1-fold of that in normal skin. Gel//HA treatment partially restored GPX4 expression, and further increases were observed in the drug-loaded groups. The complete formulation produced the strongest recovery, increasing GPX4 expression by more than tenfold relative to the model group and restoring it above the level detected in normal skin. Tacrolimus also increased GPX4 expression, although this was not accompanied by an equally pronounced reduction in total ROS.

Consistent with the impaired antioxidant defense, the model group showed extensive accumulation of 4-HNE, a product of lipid peroxidation, particularly in the epidermal and perifollicular regions (Fig. 7E). Gel//HA and Gel@bFGF/TYR//HA produced partial reductions, whereas the decrease was more pronounced in the MPDA-containing groups. Gel@bFGF/TYR//HA@MPDA reduced 4-HNE fluorescence to less than 0.1-fold of the model level, bringing it close to that observed in normal skin. The parallel reduction in ROS and 4-HNE, together with the recovery of SOD2, CAT, and GPX4, indicates that the complete formulation both relieved the cutaneous oxidative burden and reinforced endogenous antioxidant defenses.

The comparisons among formulations suggest that MPDA was a major contributor to the reduction in ROS and lipid peroxidation, consistent with its radical-scavenging activity. The additional recovery of antioxidant enzymes in the complete formulation indicates that bFGF and TYR may contribute indirectly by supporting cellular viability and tissue recovery. Consequently, Gel@bFGF/TYR//HA@MPDA treatment shifted the lesional skin from a state characterized by ROS accumulation, impaired antioxidant defense, and lipid peroxidation toward a redox profile resembling normal skin.

The concurrent reduction in ROS and 4-HNE and the recovery of SOD2, CAT, and GPX4 indicate that Gel@bFGF/TYR//HA@MPDA treatment improved the redox state of vitiligo-like skin at the tissue level. Although the changes in GPX4 and 4-HNE are consistent with attenuation of ferroptosis-associated oxidative injury, they do not independently establish ferroptosis inhibition. These tissue-level findings led us to further examine whether the antioxidant and pigmentation-associated effects were accompanied by coordinated molecular changes in redox regulation, iron metabolism, cell death, and melanogenesis.

### 3.8 Transcriptomic and protein analyses reveal coordinated remodeling of ferroptosis- and melanogenesis-related pathways

Transcriptome sequencing was performed on skin tissues collected after 3 weeks of treatment to determine whether the reductions in oxidative injury and recovery of pigmentation were accompanied by coordinated molecular changes. Differentially expressed genes between the model and Gel@bFGF/TYR//HA@MPDA groups were first subjected to pathway enrichment analysis. KEGG analysis identified significant enrichment of pathways associated with chemical carcinogenesis–reactive oxygen species, oxidative phosphorylation, autophagy, lysosomal function, glutathione metabolism, mitophagy, peroxisomes, ferroptosis, MAPK signaling, the TCA cycle, TGF-β signaling, and melanogenesis (Fig. 8A). GO analysis further identified terms related to responses to oxidative stress and hydrogen peroxide, cellular redox homeostasis, ROS metabolism, iron ion homeostasis, cellular oxidant detoxification, apoptosis, melanosomes, melanocyte differentiation, developmental pigmentation, and melanin biosynthesis (Fig. 8B). These enrichment results implicate redox regulation, iron metabolism, cell death, and pigmentation in the treatment response but do not, by themselves, indicate the direction of pathway regulation.

**Fig. 8:**
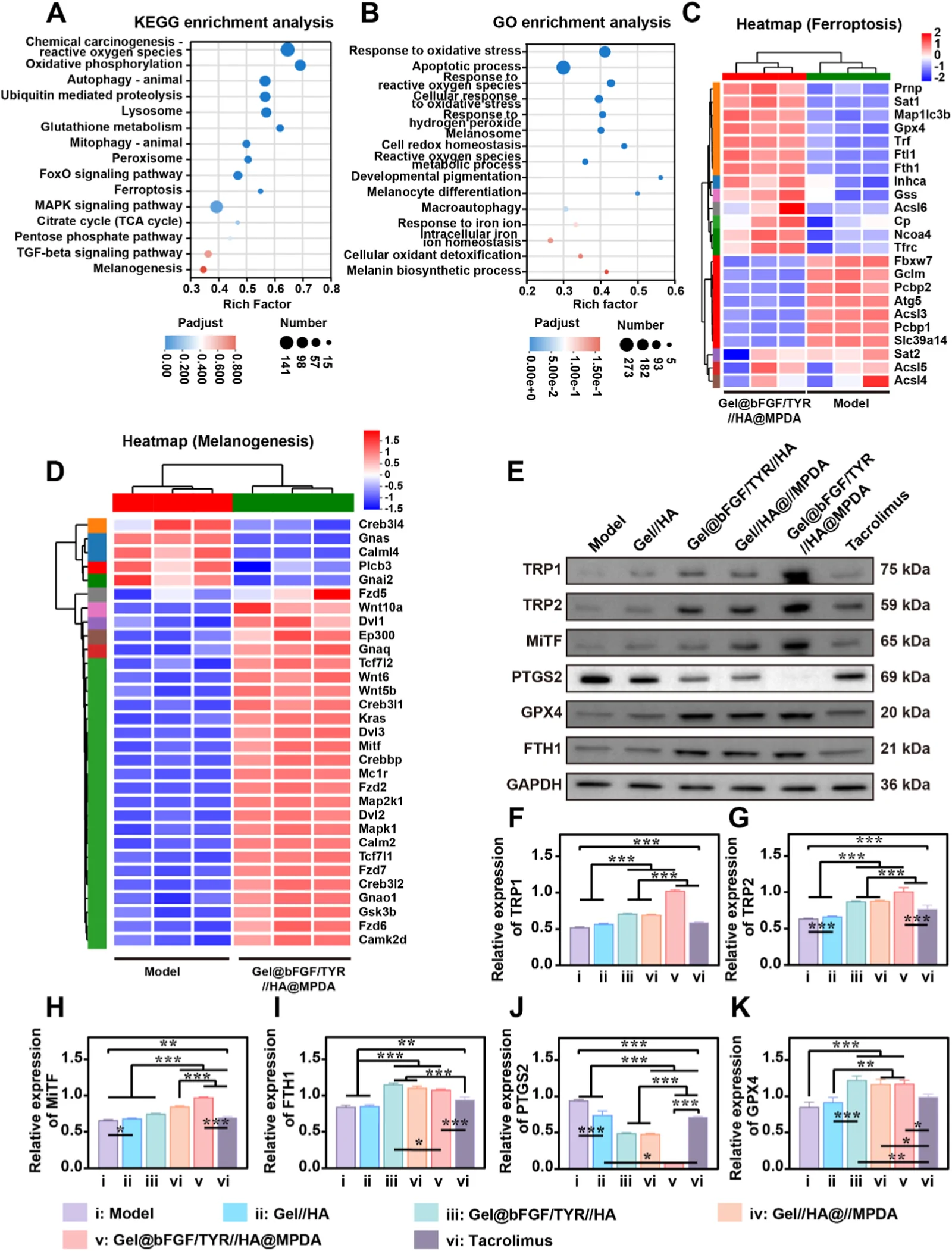
Transcriptomic analysis and protein-level validation of ferroptosis- and melanogenesis-related changes in skin tissues after microneedle treatment. (A) KEGG pathway enrichment analysis of differentially expressed genes. (B) GO enrichment analysis showing biological processes associated with oxidative stress, antioxidant regulation, cell death, melanosome organization, and melanocyte function. Heatmaps of differentially expressed genes associated with (C) ferroptosis and (D) melanogenesis-related signaling. (E) Representative Western blot images of GPX4, FTH1, TRP1, TRP2, MITF, PTGS2, and GAPDH. Quantitative analysis of the melanogenesis-related proteins (F) TRP1, (G) TRP2, and (H) MITF, and the ferroptosis-related proteins (I) FTH1, (J) PTGS2, and (K) GPX4.

The ferroptosis-related heatmap clearly separated the Gel@bFGF/TYR//HA@MPDA and model samples, indicating substantial remodeling of this gene set following treatment (Fig. 8C). Several changes were consistent with enhanced resistance to oxidative and lipid peroxidative injury. The antioxidant defense genes *Gpx4* and *Gss* were upregulated, as were the iron-storage genes *Fth1* and *Ftl1*. Increased expression of *Trf* and *Cp* further indicated altered iron transport and handling. Conversely, *Acsl4*, which promotes the incorporation of polyunsaturated fatty acids into phospholipids susceptible to peroxidation, was downregulated in the treatment group.

The transcriptional pattern was not uniformly indicative of ferroptosis inhibition. *Sat1*, *Tfrc*, and *Ncoa4* were increased following treatment, whereas *Gclm* and *Acsl3* were reduced. In particular, ACSL3-dependent monounsaturated fatty acid metabolism has been reported to confer resistance to ferroptosis; therefore, its downregulation cannot be interpreted as evidence of reduced ferroptotic susceptibility [44]. The heatmap consequently supports treatment-associated remodeling of ferroptosis-related antioxidant and iron-handling networks rather than a simple, unidirectional suppression of the entire pathway.

Western blotting was used to examine whether selected transcriptomic changes were reflected at the protein level (Fig. 8E). Compared with the model group, the complete formulation increased GPX4 and FTH1 expression while reducing PTGS2 expression (Fig. 8I–K). The increase in GPX4 was particularly evident in the MPDA-containing and complete formulation groups, consistent with the recovery of GPX4 immunofluorescence observed in skin tissues. The protein-level changes were also consistent with the reductions in ROS and 4-HNE shown in Fig. 7, supporting enhanced antioxidant defense and attenuation of ferroptosis-associated oxidative injury. Because ferroptotic cell death was not directly measured, however, these molecular changes should not be regarded as definitive evidence that ferroptosis was inhibited.

The melanogenesis-related heatmap showed a distinct separation between the model and Gel@bFGF/TYR//HA@MPDA groups (Fig. 8D). Most genes involved in melanogenic signaling were expressed at lower levels in the model group and were upregulated following treatment. These included the Wnt ligands *Wnt5b*, *Wnt6*, and *Wnt10a* and the Frizzled receptors *Fzd2*, *Fzd5*, *Fzd6*, and *Fzd7*. Intracellular signaling mediators, including *Dvl1*, *Dvl2*, *Dvl3*, *Map2k1*, *Mapk1*, *Camk2d*, and *Calm2*, also showed higher expression in the treatment group. This pattern indicates treatment-associated recovery of Wnt/Frizzled-, MAPK-, and Ca^2+^-related signaling networks involved in melanocyte regulation and pigmentation [45], [46], [47].

Consistent with these changes, *Mitf*, a central regulator of melanocyte differentiation and melanogenic activity, was upregulated together with the transcriptional co-regulators *Ep300* and *Crebbp* (Fig. 8D). Increased expression of *Mc1r*, *Gnaq*, and *Gnao1* further supported recovery of upstream signaling associated with melanocyte function. At the protein level, Western blotting showed increased expression of MITF and its downstream melanogenic proteins TRP1 and TRP2 (Fig. 8E). Densitometric analyses confirmed that the complete Gel@bFGF/TYR//HA@MPDA formulation produced the highest expression of TRP1, TRP2, and MITF among the treatment groups (Fig. 8F–H, respectively). The transcriptional recovery of melanogenesis-related signaling was therefore accompanied by corresponding changes in melanogenic protein expression.

Previous studies provide a biological basis for the simultaneous changes in redox regulation and melanogenesis observed here. In primary human epidermal melanocytes, erastin-induced oxidative stress, iron accumulation, and lipid peroxidation were accompanied by reduced melanin synthesis, whereas these alterations were attenuated by N-acetyl-L-cysteine treatment [5]. The Wnt/β-catenin–MITF pathway has also been linked to ferroptosis sensitivity through downstream regulators such as PGC1α and SCD1 in melanocytic-lineage melanoma models [48]. In addition, downregulation of ferritin light chain was reported to impair tyrosinase maturation and induce depigmentation in human metastatic melanoma cells, suggesting that iron storage and pigment production may be functionally connected [49].

The present results show that Gel@bFGF/TYR//HA@MPDA treatment was associated with enhanced antioxidant defense, altered iron-handling pathways, reduced ferroptosis-associated molecular features, and recovery of MITF-related melanogenic signaling. These molecular changes agree with the lower ROS and 4-HNE levels and increased tissue pigmentation observed *in vivo*. They do not establish that attenuation of ferroptosis directly caused the recovery of MITF signaling or melanin production, because both responses may have occurred in parallel as the oxidative microenvironment improved. Establishing such a causal relationship will require ferroptosis-specific pharmacological intervention and direct measurements of lipid ROS, labile iron, and ferroptotic cell death.

## 4 Conclusion

In this study, we developed a bilayer Gel//HA microneedle system in which MPDA was incorporated into the rapidly dissolving HA compartment, while bFGF and TYR were loaded into the crosslinked GelMA/HAMA layer for sustained release. The complete formulation reduced oxidative injury and supported the proliferation and migration of HaCaT cells, while restoring redox balance and increasing melanin-associated content in B16-F10 cells. In monobenzone-induced vitiligo-like lesions, Gel@bFGF/TYR//HA@MPDA promoted repigmentation, reduced CD8, IFN-γ, and TNF-α signals, restored antioxidant defenses, and limited ROS and 4-HNE accumulation. Transcriptomic and protein analyses further showed attenuation of ferroptosis-associated molecular alterations and recovery of MITF-related melanogenic signaling. These results support the bilayer microneedle system as a localized strategy for simultaneously improving the oxidative, inflammatory, and pigmentation-associated features of vitiligo.

## CRediT authorship contribution statement

**Jingyao Liang:** Writing – original draft, Methodology, Investigation, Funding acquisition, Formal analysis. **Wenyan Liu:** Validation, Software, Methodology, Investigation, Data curation. **Yu Jiang:** Visualization, Validation, Investigation, Conceptualization. **Yihui Yu:** Resources, Investigation. **Qian Li:** Validation, Data curation. **Yuyan Zhang:** Visualization, Software, Funding acquisition. **Wei Li:** Writing – review & editing, Supervision, Project administration, Conceptualization. **Xibao Zhang:** Writing – review & editing, Supervision, Project administration, Conceptualization.

## Ethics approval and consent to participate

The Zhuhai BesTest Bio-Tech Co., Ltd for the Care and Use of Laboratory Animals was followed for all animal experiments (IAC25W039).

## Funding

This study was supported by Guangzhou Basic Research Plan Jointly Funded by the City, School (Hospital), and/or Enterprise (Grant No. 2023A03J0944, and 2023A03J0678); the Scientiffc Research Project of Guangdong Provincial Bureau of Traditional Chinese Medicine (Grant No. 20261300); the Medical Science and Technology Research Foundation of Guangdong Province (Grant No. A2026076).

## Declaration of competing interest

The authors declare that they have no known competing financial interests or personal relationships that could have appeared to influence the work reported in this paper.

## Data Availability

Data will be made available on request.
