# Supplementary material for: Microneedle-mediated co-delivery of biomimetic melanin and pro-regenerative factors for oxidative and immune modulation in vitiligo
Source: Mater Today Bio. 2026 Oct 17;41:103757. doi: 10.1016/j.mtbio.2026.103757 (PMC13698074; doi:10.1016/j.mtbio.2026.103757)
Supplement: Supplementary file 1 [file mmc1.docx]

**Microneedle-Mediated Co-Delivery of Biomimetic Melanin and Pro-Regenerative Factors for Oxidative and Immune Modulation in Vitiligo**

Jingyao Liang^1,2^‡, Wenyan Liu^1,2^‡, Yu Jiang^1,2^‡, Yihui Yu^1,2^, Qian Li^1,2^, Yuyan Zhang^1,2^, Wei Li^1,2^*_,_ Xibao Zhang^1,2^*

^1^Institute of Dermatology, Guangzhou Medical University, Guangzhou 510095, P.R. China

^2^Department of Dermatology, Guangzhou Dermatology Hospital, Guangzhou 510095, P.R. China

‡These three authors contributed equally to this work.

*Correspondence should be addressed to:

Xibao Zhang, E-mail: [gzpfbfzs@163.com](mailto:gzpfbfzs@163.com)

Wei Li, E-mail: 907327637@qq.com

Department of Dermatology, Guangzhou Dermatology Hospital, 56 Hengfu Road, Guangzhou 510095, P.R. China

Tel.: 8620-83593476

**Fig. S1. Schematic illustration of the fabrication of blank and drug-loaded double-layer hydrogel microneedles.** (A) Fabrication of Gel//HA microneedles. The Gel precursor solution was cast into the microneedle mold and photocrosslinked to form the outer Gel hydrogel shell, followed by the addition and drying of the HA solution to form the inner layer. (B) Fabrication of Gel@bFGF/TYR//HA@MPDA microneedles. The bFGF/TYR-loaded Gel precursor solution was photocrosslinked to form the outer hydrogel shell, after which the MPDA-containing HA solution was added and dried to generate the MPDA-loaded inner layer.

**Fig. S2. Morphological and size characterization of MPDA released from Gel@bFGF/TYR//HA@MPDA microneedles.**

**Fig. S3. Concentration-dependent cytocompatibility of MPDA, bFGF, and TYR in HaCaT cells.** Cell viability of HaCaT cells after 24 h of exposure to different concentrations of (A) MPDA, (B) bFGF, and (C) TYR, as determined by the CCK-8 assay. n.s., not significant; *P < 0.05, **P < 0.01, and ***P < 0.001.

**Fig. S4. CCK-8 assay of cell viability after HaCaT cells were treated with different concentrations of H_2_O_2_.**

**Fig. S5. Effects of different hydrogel microneedle extracts on inflammatory protein expression in H_2_O_2_-injured HaCaT cells.** (A) Representative Western blot images of IFN-γ, TNF-α, and GAPDH. (B) Quantitative analysis of IFN-γ and TNF-α protein expression.

**Fig. S6. Effects of TYR/bFGF and MPDA, alone or in combination, on B16-F10 cell viability and melanin content.** (A) Viability of B16-F10 cells after treatment with TYR/bFGF, MPDA, or their combination for 1, 2, and 3 days, as determined by the CCK-8 assay. (B) Melanin content of B16-F10 cells after treatment with the different formulations for 24 h. *P < 0.05, **P < 0.01, and ***P < 0.001.
